# Recombination of chl-*fus* gene (Plastid Origin) downstream of *hop*: a locus of chromosomal instability

**DOI:** 10.1186/s12864-015-1780-1

**Published:** 2015-08-04

**Authors:** Libia Catalina Salinas Castellanos, Jacques Chomilier, Jorge Hernández-Torres

**Affiliations:** Laboratorio de Biología Molecular, Escuela de Biología, Universidad Industrial de Santander, Apartado Aéreo 678, Bucaramanga, Colombia; IMPMC, UPMC, CNRS UMR 7590, MNHN, IRD, Paris, France and RPBS, Paris, France

**Keywords:** TPR proteins, *hop* gene, cEF-G, chl-*fus* gene, Microsynteny, Exon shuffling, Intron phase

## Abstract

**Background:**

The co-chaperone Hop [*h*eat shock protein (HSP) *o*rganizing *p*rotein] has been shown to act as an adaptor for protein folding and maturation, in concert with Hsp70 and Hsp90. The *hop* gene is of eukaryotic origin. Likewise, the chloroplast elongation factor G (cEF-G) catalyzes the translocation step in chloroplast protein synthesis. The chl-*fus* gene, which encodes the cEF-G protein, is of plastid origin. Both proteins, Hop and cEF-G, derived from domain duplications. It was demonstrated that the nuclear chl-*fus* gene locates in opposite orientation to a *hop* gene in *Glycine max*. We explored 53 available plant genomes from Chlorophyta to higher plants, to determine whether the chl-*fus* gene was transferred directly downstream of the primordial *hop* in the proto-eukaryote host cell. Since both genes came from exon/module duplication events, we wanted to explore the involvement of introns in the early origin and the ensuing evolutionary changes in gene structure.

**Results:**

We reconstructed the evolutionary history of the two convergent plant genes, on the basis of their gene structure, microsynteny and microcolinearity, from 53 plant nuclear genomes. Despite a high degree (72 %) of microcolinearity among vascular plants, our results demonstrate that their adjacency was a product of chromosomal rearrangements. Based on predicted exon − intron structures, we inferred the molecular events giving rise to the current form of genes. Therefore, we propose a simple model of exon/module shuffling by intronic recombinations in which phase-0 introns were essential for domain duplication, and a phase-1 intron for transit peptide recruiting. Finally, we demonstrate a natural susceptibility of the intergenic region to recombine or delete, seriously threatening the integrity of the chl-*fus* gene for the future.

**Conclusions:**

Our results are consistent with the interpretation that the chl-*fus* gene was transferred from the chloroplast to a chromosome different from that of *hop*, in the primitive photosynthetic eukaryote, and much later before the appearance of angiosperms, it was recombined downstream of *hop*. Exon/module shuffling mediated by symmetric intron phases (i.e., phase-0 introns) was essential for gene evolution. The intergenic region is prone to recombine, risking the integrity of both genes.

**Electronic supplementary material:**

The online version of this article (doi:10.1186/s12864-015-1780-1) contains supplementary material, which is available to authorized users.

## Background

Conserved synteny is the degree to which genes remain on corresponding chromosomes [1, 2]. The analysis of conserved microsynteny (i.e., small regions of synteny) is a useful method to unveil the molecular events that have occurred since the transfer of organellar genes to the nucleus. To unravel the details of genome recombination during speciation that are associated with the formation of new species, conserved microsynteny analysis is also essential. Otherwise, gene colinearity is the conservation of gene content and orders over time [1]. The study of how gene orders are conserved reveals the degree of chromosome rearrangement within specific genomes. In this work, we describe the evolutionary history of two convergent plant transcription genes, *hop* and chl-*fus*. We examined the gene microsynteny and microcolinearity of the pair *hop* (nuclear origin) – chl-*fus* (chloroplast origin) from 53 plant nuclear genomes, describe their phylogenetic relationships, and discuss the influence of intron phase distribution on the evolution of both genes by exon shuffling. Predicted recombination events, in higher plants, support the hypothesis that the chromosomal region downstream of the *hop* gene is prone to recombine, having favored the shuffling of the chloroplast chl-*fus* gene adjacently to *hop*, in an opposite orientation.

The co-chaperone Hop [*h*eat shock protein (HSP) *o*rganizing *p*rotein] has been shown to bind both Hsp70 and Hsp90 into supercomplexes that act as an adaptor for protein folding and maturation [3]. The Hop protein is composed of three TPR domains: TPR1 is followed by one DP domain and then one Ch. AA (charged amino acids) domain; TPR2A; and TPR2B, which is followed by one DP domain [4, 5]. Previous analyses of human and mouse genomes suggest that *hop* genes result from successive duplication of an ancestral TPR–DP module surrounded by introns of the same phase [6]. Hop is a ubiquitous eukaryotic protein, implying that its evolutionary origin dates back to the emergence of the first eukaryotic cells [7]. Furthermore, molecular and bioinformatics studies conclude that Hop is encoded by orthologous gene families in all eukaryotes [6]. The role of the *hop* gene in plants has not been well established but mRNA expression was induced under stress conditions [8]. The *hop* gene is also found in plants; one member of the family was found in *Glycine max*, downstream in convergent transcription with the chl-*fus* gene, which encodes the chloroplast-specific translation elongation factor G (cEF-G) [8]. The elongation factor G exhibits two main functions: it catalyzes the translocation step of bacterial, mitochondrial and chloroplast protein synthesis [9, 10], and together with ribosome recycling factor (RRF), it promotes the disassembly of the post-termination ribosome [11]. The chl-*fus* gene was horizontally transferred from the primitive chloroplasts to the nucleus of the first photosynthetic eukaryotes [12]. Thus, the fact that chl-*fus* lies in the 3′ flanking region of a very ancient gene like *hop* leads to hypothesize, that probably chl-*fus* was originally inserted − among other potential sites − in this site. The conservation of the microsynteny and microcolinearity of the pair of convergent genes needed to be verified in order to clarify the reason of the successful gene transfer of a functional chl-*fus* to the nucleus, among many attempts that may have occurred.

According to the endosymbiotic theory, chloroplasts and mitochondria arose from the engulfment of prokaryotic cells by a proto-eukaryotic cell. Through evolutionary time, around 14-20 % of genes of chloroplast genome were transferred to the nucleus [13–15]. As a consequence, the transferred genes had to adapt to the nuclear genetic system (i.e., eukaryotic promoters, spliceosomal introns, etc.). Nuclear-encoded chloroplast proteins that are synthesized in the cytosol are imported through the outer and inner envelope membranes of chloroplast; this is possible because transferred genes recruited DNA sequences coding for an N-terminal transit peptide [16]. From the sequencing of the first plastid genomes e.g., *Nicotiana tabacum* [17], *Marchantia polymorpha* [18], *Oryza sativa* [19], *Euglena gracilis* [20], it was concluded that the chl-*fus* gene is no longer located in the chloroplast but strictly found in the nucleus [21]. The first plant chl-*fus* gene was cloned and sequenced from *Glycine max*; it is split three times by introns of 330, 508 and 288 bp [12]. The first exon codes for a typical chloroplast transit peptide that must be removed after translocation into the stroma [16]. Surprisingly, near to nothing has been published about the plant chl-*fus* gene, since it was cloned and sequenced in *G. max* [12], specifically on the regulation of its expression.

The microcolinearity between *hop* and chl-*fus* genes in *G. max* raises many interesting questions: are all *hop* and chl-*fus* plant genes arranged in a convergent orientation, as in *G. max* (microcolinearity)? Was chl-*fus* directly transferred from chloroplasts, downstream of the primordial *hop*? If that were the case, would it be possible to explain, based on sequence analysis, why the chl-*fus* gene was not successfully transferred and functionally established in a location different of the actual one? In vertebrates, the *hop* gene is organized in recombinable TPR − DP modules, surrounded by introns of the same phase. This could explain the evolutionary origin of *hop* by triplication of an ancient TPR − DP unit. Does the exon–intron organization of plant *hop* genes support this hypothesis? And finally, how can the study of the pair of genes *hop* and chl-*fus* contribute to the understanding of the evolution of plant genomes? Here, all these questions are discussed and, on the basis of the findings, models for the evolution of *hop* and chl-*fus* genes are proposed.

## Results

### Capture and validation of plant *hop* and chl-*fus* gene sequences

The first chl-*fus* gene was cloned and characterized in *G. max* [12]. From protein sequence alignments of its encoded open reading frame (ORF), as well as chloroplast-type transit peptide analysis, it was suggested that the mature protein belongs to the chloroplast protein synthesis machinery [12, 22]. For example, the *Arabidopsis thaliana* cEF-G (At_cEF-G) shares 44 % identity with its mitochondrial counterpart (At_mEF-G), while 59 % with *Escherichia coli* EF-G (γ-Proteobacteria), 54 % with *Synechococcus* sp. EF-G (Cyanobacteria) and 62 % with *Agrobacterium fabrum* (α-Proteobacteria) EF-G. Many other chl-*fus* genes have been registered in Genbank, sometimes confounded with mEF-G (not shown).

Gene mapping efforts in *G. max,* following the discovery of chl-*fus* gene, revealed that chl-*fus* locates downstream of *hop* gene in an opposite orientation [8]. Microsynteny analyses of new sequenced genomes would help us to determine if the transcriptional convergence of *hop* and chl-*fus* genes is ubiquitous, or if *G. max* is an isolated case. We then used the *G. max* chl-*fus* gene as a BLAST query sequence to search for plant genomic contigs, coding for a predicted cEF-G preceded by a chloroplast-type transit peptide [12], concurrently with a *hop* gene in convergent transcription. The families, genera and species, and corresponding accession numbers of retrieved contigs obtained from Genbank are provided in Table 1. In plant species whose chl-*fus* and *hop* genes were not syntenic, the *G. max hop* gene alone [8] was used as query to capture Hop encoding sequences. Using the *G. max* chl-*fus* and *hop* genes as references, we mapped the predicted exon–intron structure of each gene for all plant species. To validate the assembled ORFs, phylogenetic trees were constructed *in silico* with predicted cEF-G and Hop proteins.

**Table 1:** Accession numbers of retrieved contigs sequences obtained from plant genome databases. The number of introns of *hop* and chl-*fus* genes, respectively, is given in arabic numbers

Family	Species	Introns	Genbank Accession numbers
CHLOROPHYTA			
Mamiellaceae	*Micromonas sp. RCC299*	1-1	XP_002500383; XP_002500081
	*Ostreococcus lucimarinus*	0-1	XP_001418158; XP_001419031
	*Ostreococcus tauri*	0-1	XM_003079642; XM_003080500
Chlamydomonadaceae	*Chlamydomonas reinhardtii*	12-9	XP_001691869; XM_001701793
GYMNOSPERMS			
Funariaceae	*Physcomitrella patens*	8-6	NW_001865607; XP_001784483
Pinaceae	*Picea abies*	8-3	MA_10426940; MA_10431292(*)
			(*) Dendrome Project
MONOCOTS			
Musaceae	*Ensete ventricosum*	6-3	AMZH01008475; AMZH01015354
Poaceae	*Brachypodium distachyon*	6-3	NC_016135
	*Oryza glaberrima*	6-3	ADWL01008993
	*Oryza sativa*	6-3	CM000129
	*Setaria italica*	6-3	NW_004675967
	*Sorghum bicolor*	6-3	NC_012875
	*Zea mays*	6-3	GK00032
Arecaceae	*Elaeis guineensis*	6-3	ASJS01002389-94
	*Phoenix dactylifera*	6-3	ATBV01012962
DICOTS			
Cucurbitaceae	*Citrullus lanatus*	6-3	AGCB01004585; AGCB01006484
	*Cucumis melo*	6-3	CAJI01012439; CAJI01003926
	*Cucumis sativus*	6-3	XM_004147890; XM_004147564
Cannabaceae	*Cannabis sativa*	6-3	AGQN01077260
Moraceae	*Morus notabilis*	6-3	ATGF01007958
Rosaceae	*Fragaria vesca subsp vesca*	6-3	NC_020495
	*Malus domestica*	6-3	ACYM01058960
	*Prunus mume*	6-3	AOHF01010810
	*Prunus persica*	6-3	AEKV01005456
	*Pyrus x bretschneideri*	6-3	AJSU01026097
Fabaceae	*Cajanus cajan*	6-3	AGCT01009484-85
	*Cicer arietinum*	6-3	XM_00451602; XM_004515686
	*Glycine max*	6-3	XP_003549898
	*Lupinus angustifolius*	6-3	AOCW01121688; AOCW01054016
	*Medicago truncatula*	6-3	NC_016411; NC_016410
Euphorbiaceae	*Hevea brasiliensis*	6-3	AJJZ010763885
	*Jatropha curcas*	6-3	BABX02001448
	*Ricinus communis*	6-3	NW_002994274
Linaceae	*Linum usitatissimum*	6-3	AFSQ01027627-29
Salicaceae	*Populus trichocarpa*	6-3	NC_008469
Malvaceae	*Gossypium raimondii*	6-3	AMOP01022205
	*Theobroma cacao*	6-3	CACC01007881
Brassicaceae	*Aethionema arabicum*	5-3	ASZG01007785
	*Arabidopsis lyrata*	6-3	NW_003302554
	*Arabidopsis thaliana*	6-3	NC_003070
	*Brassica rapa*	6-2	AENI01007476
	*Capsella rubella*	6-3	ANNY01000463
	*Eutrema parvulum*	6-3	AFAN01000006
	*Eutrema salsugineum*	6-3	AHIU01002482
	*Leavenworthia alabamica*	6-3	ASXC010000179
	*Sisymbrium irio*	6-3	ASZH01019437
Caricaceae	*Carica papaya*	6-3	ABIM01007984
Rutaceae	*Citrus sinensis*	6-3	AJPS01000059
Vitaceae	*Vitis vinifera*	6-3	AM459130
Solanaceae	*Nicotiana sylvestris*	6-3	ASAF01010839-40
	*Nicotiana tomentosiformis*	6-3	ASAG01110979
	*Solanum lycopersicum*	6-3	AP009300
	*Solanum tuberosum*	6-3	AEWC01024049

(*) mean that MA_10426940 and MA_10431292 sequences were retrieved from Dendrome Project

**Fig. 1:**
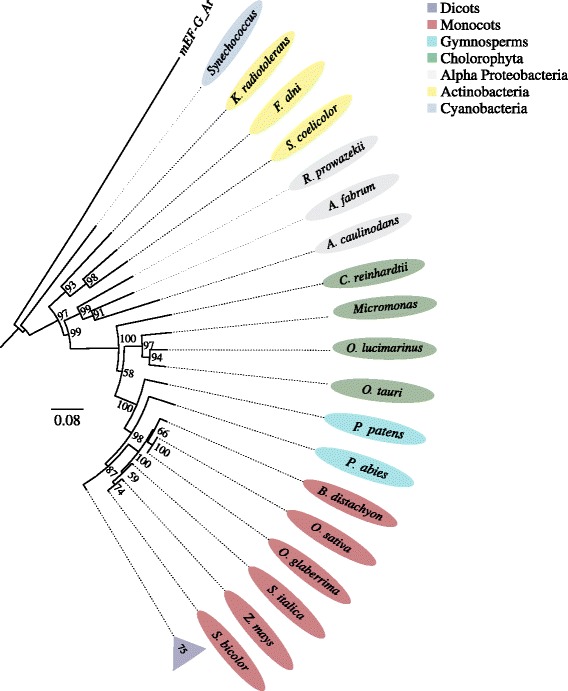
Phylogenetic tree of chloroplast elongation factor cEF-G sequences from 53 plant genomes. Bootstrap values are in Arabic numbers. Dicot branch was collapsed (bootstrap values less than 50 %). Other members of the EF-G family: At_mEF-G: *A. thaliana* mitochondrial elongation factor G (outgroup). α-Proteobacterial EF-G: *R. prowazekii*, *A. caulinodans* and *A. fabrum*. Actinobacterial EF-G: *K. radiotolerans*, *F. alni* and *S. coelicolor*. Cyanobacterial EF-G: *Synechococcus*. 0.08: Distance scale

We show in Fig. 1 a well-supported phylogenetic tree constructed with EF-G sequences from Actinobacteria, α-Proteobacteria and Cyanobacteria and 53 cEF-G sequences from Chlorophyta, Gymnosperms, Monocots and Dicots. The branching pattern of the cladogram indicates that EF-Gs from all life forms descended from a common ancestor. According to the evolutionary relationships, plant cEF-G sequences group together in a single branch with *G. max* cEF-G (our reference sequence), confirming that the assembled plant ORFs belong all to the chloroplast EF-G family. Chlorophyta cEF-G sequences share a common ancestor with higher plants, excepting *Chlamydomonas reinhardtii*, which appears to form a clade apart from other members of green algae. The two gymnosperms are part of the major clade with vascular plants although in separate lineages. Monocot and dicot branches are coherent with canonical evolutionary trees; however, dicot branch had low support (bootstrap values less than 50 %) resulting in this clade being unresolved [23]. As already reported [10], cEF-G sequences show more identity with α-proteobacterial EF-G than with cyanobacteria and this finding is confirmed in Fig. 1, without exception. Taking these results together, we concluded that retrieved cEF-G sequences from Genbank were correctly reconstructed and they code for the chloroplast translation elongation factor G.

**Fig. 2:**
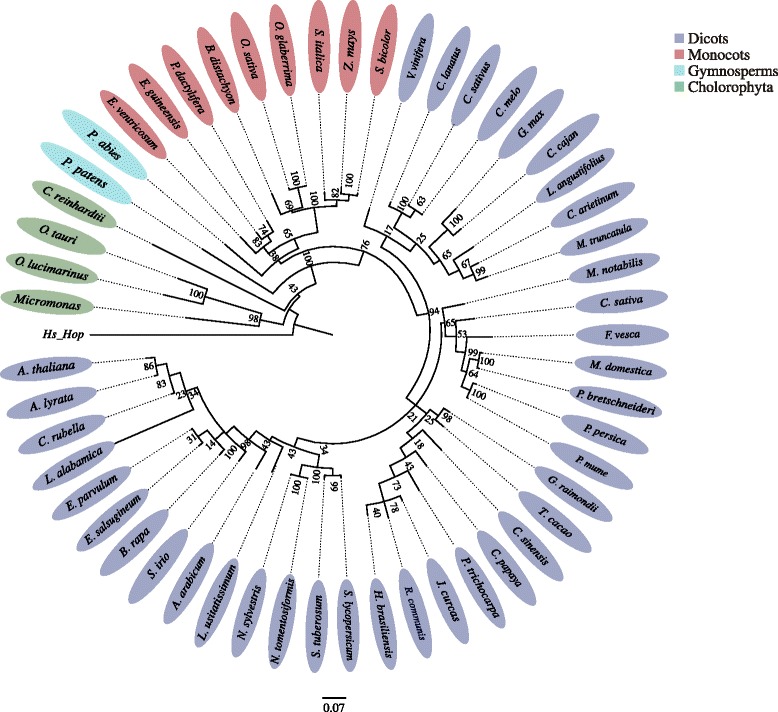
Phylogenetic tree of Hop protein sequences of 53 plant genomes. Chlorophyta, gymnosperm, monocot and dicot orthologous proteins were included. Hs_Hop: Human Hop protein (outgroup). 0.07: Distance scale

After intron removal from *hop* genes, the reconstructed Hop sequences were used to build a second phylogenetic tree (Fig. 2). As expected, the assembled ORFs belong all to the plant Hop family which exhibits a large amount of divergence with respect to the outgroup (Human Hop). As seen in Fig. 2 the inferred relationships among these protein sequences are robust and all branches are well supported, coherently with current plant systematics.

Interestingly, *Leavenworthia alabamica* is grouped with the other members of Brassicales but with an unusual long evolutionary distance (Fig. 2). Exceptionally, *L. alabamica* contains three tandem repetitions of the VPEVEKKLEPEPEP motif within the Ch. AA domain, while all other plants possess only one. These results confirm the correct assembly of *hop* genes from retrieved contigs.

### Preserved microsynteny and microcolinearity between *hop* and chl-*fus* genes

**Fig. 3:**
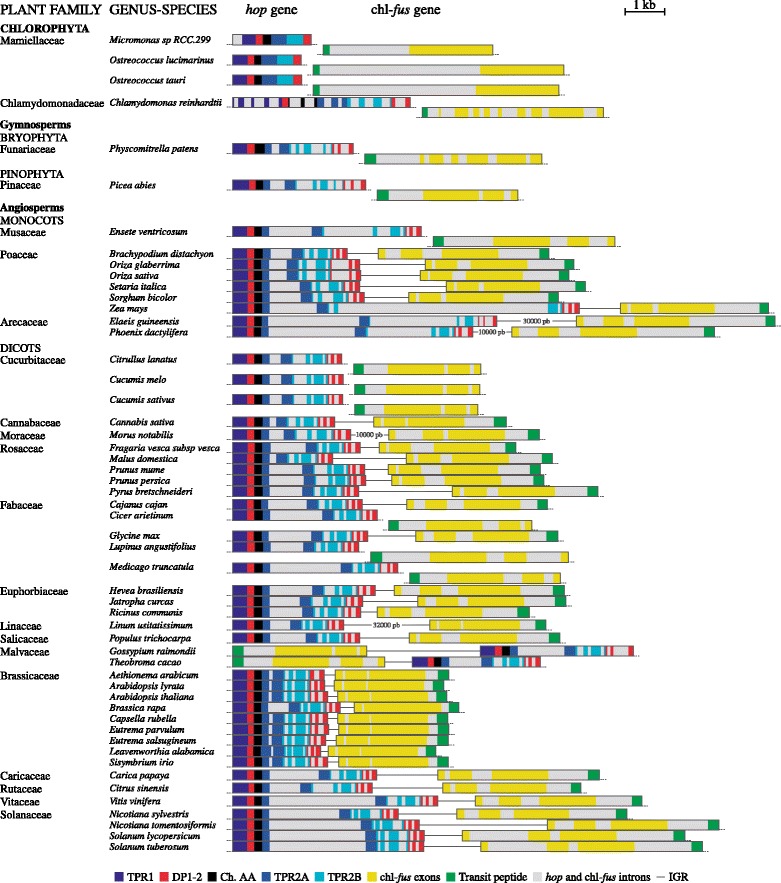
Microsyntenic arrangement (at scale) of the pair of genes *hop* and chl-*fus,* among the 53 plant genomes under study. Hop protein TPR and DP domains are color-coded according to conventions (bottom boxes). IGR: intergenic region. IGR containing numbers, e.g., 10000 bp, are not at scale. Non-syntenic genes are drawn on separate chromosomes

The *hop* and chl-*fus* genes were discovered in *G. max* one after the other on the same chromosome, in convergent transcription arrangement [8]. This finding leads to two intriguing evolutionary questions: Have *hop* and chl-*fus* genes been together from the first to the present-day photosynthetic eukaryotes? Or, is their chromosomal contiguity strictly specific of *G. max*? The microsyntenic arrangement of *hop* and chl-*fus* genes was determined for all 21 plant families under study (Fig. 3, and species-specific details in Additional file 1: Figure S1). In Clorophyta, two families were mapped (Mamiellaceae and Chlamydomonadaceae) and each gene was found on a separate chromosome, suggesting the absence of microsynteny in this plant division. This was also the case for gymnosperms (Funariaceae and Pinaceae). In return, 2 out of 3 studied families of monocots revealed the presence of *hop* and chl-*fus* genes on the same chromosome. Only in *Ensete ventricosum* (Musaceae), the pair of genes was found on separate chromosomes. In the same manner, the microsynteny is preserved in most of dicots excepting the Cucurbitaceae (3 species) and Fabaceae (3 out of 5 species) families, where the pair of genes is located on different chromosomes (Additional file 2: Table S1). In summary, the microsynteny of *hop* and chl-*fus* prevails in 75 % (40 out of 53) of green plants studied. A graphic resume of microsynteny between *hop* and chl-*fus* genes among all plant species under study is shown in Additional file 3: Figure S2.

**Fig. 4:**
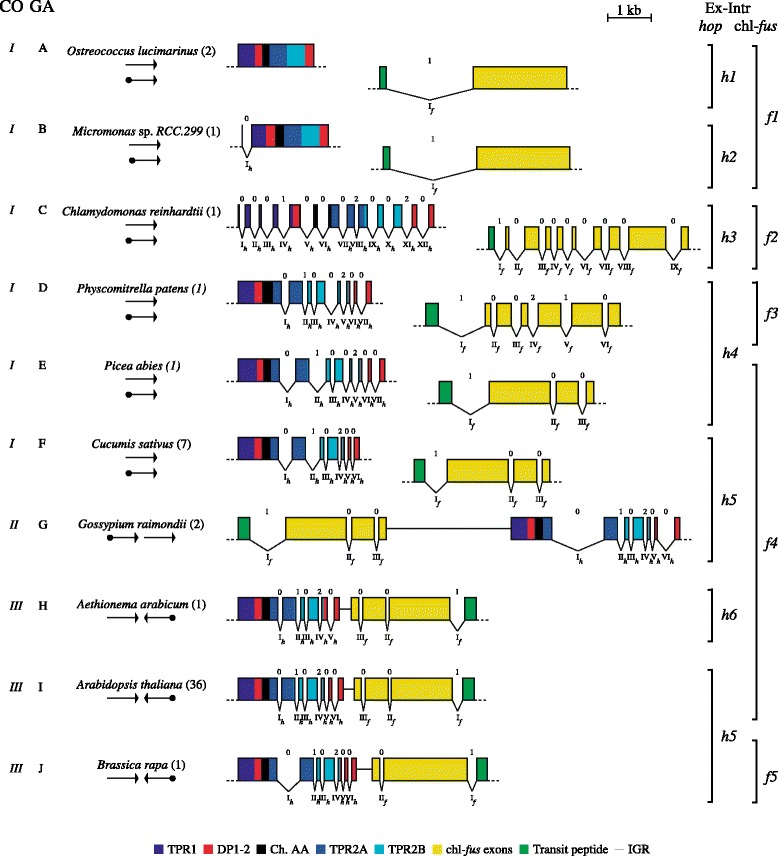
Grouping of gene arrangements found for the pair of genes *hop* and chl-*fus,* among the 53 plant genomes under study. CO: classification by microcolinearity (categories *I* to *III*); GA: classification by gene arrangement, according to the exon–intron structure of both combined *hop* and chl-*fus* (categories A to J). Arabic numbers in parenthesis: number of species sharing the same gene arrangement; *hop* and chl-*fus* genes are represented by arrows to resume gene topology. Ex-Intr *hop*: exon–intron organizations found for *hop* gene (categories *h1* to *h6*), Ex-Intr chl-*fus*: exon–intron organizations found for chl-*fus* gene (categories *f1* to *f5*). Arabic and roman numbers represent intron phase (0, 1, or 2) and succession of introns from I to I + n, respectively; *hop* introns are named as I_*h*_, II_*h*_, III_*h*_, etc., and chl-*fus* introns are named as I_*f*_, II_*f*_, III_*f*_, etc. Exons coding for TPR and DP domains are color-coded according to conventions (bottom boxes). IGR: intergenic region. Non-syntenic genes are drawn on separate chromosomes

Concerning the one-to-one microcolinearity in convergent transcription of *hop* and chl-*fus*, three types of genome arrangements (*I* to *III*) were found in plants (Fig. 4), as follows: *I*). Each gene resides on a different chromosome, i.e., they are not collinear (all Chlorophyta, gymnosperms, one monocot, and six dicots). *II*) In Malvaceae (*Gossypium raimondii* and *Theobroma cacao*) the chl-*fus* gene moved just upstream of *hop* and both genes are transcribed in the same direction, i.e., local chromosome inversion [24, 25]; and *III*) *hop* and chl-*fus* are colinear in convergent transcription (no inserted elements), which is the most frequent arrangement in both monocots and dicots (38 out of 53 species analyzed or ≈ 72 %). Interestingly, *Elaeis guineensis* and *Phoenix dactylifera* (monocots), as well as *Morus notabilis* and *Linum usitatissimum* (dicot) harbored sequences coding for retrovirus-like proteins within their intergenic sequences, i.e., inserted between *hop* and chl-*fus* genes (see the section about molecular instability of the intergenic region). Detailed physical maps for each species under study are shown in Additional file 1: Figure S1.

### Parallel evolution of exon–intron gene structure of *hop* and chl-*fus* genes

The human *hop* gene contains 13 introns and intron phase was essential to hypothesize the evolutionary origin of Hop domains, by exon shuffling [6]. However, intron number and phase of plant *hop* genes are still unknown and this data could reinforce the role of introns in *hop* evolution from the initial stages of eukaryotic development. Therefore, we examined the exon–intron organization of *hop* and chl-*fus* genes among the 53 plant species, to infer the contribution of introns to the evolution of their resultant proteins (Table 1, Fig. 3, 4 and Additional file 1: Figure S1).

The simultaneous spatial arrangement of exons and introns in the coding sequences of the pair *hop* − chl-*fus* in plants falls in one of ten categories (A to J), as shown in Fig. 4. In type A (*O. lucimarinus* and *O. tauri*), *hop* lacks introns, while chl-*fus* holds a single intron splitting the mature protein from the transit peptide-coding exon (labelled as I_*f*_). Apparently, *Micromonas* sp. does not contain introns; however, it is very likely that a 5′ intron is located after the first 18 nucleotides. An exceptionally long predicted Hop protein is reported in Genbank under the accession number XP_002500383; this polypeptide shares high identity with other plant Hop proteins, but contains 71 extra amino acids not found in any other eukaryote. A fine-scale analysis of this insertion suggests that an intron may have gone unnoticed so far because it is in frame with a 5′ short exon, coding for the conserved amino acids MADEHK. We show in Additional file 4: Figure S3 (A) an HCA alignment of predicted *Micromonas* sp. [GenBank: XP_002500383] with *A. thaliana* Hop proteins. In this alignment, a perfect match is obvious between the two proteins, excluding the extra 71 N-terminal amino acids of *Micromonas* sp. (bordered by a rounded rectangle). In Additional file 4: Figure S3 (B), we represent the translated 5′ regions of *Micromonas* sp. and predicted *C. reinhardtii hop* genes. We propose that nucleotides in bold belong to a phase-0 intron (I_*h*_), which is in frame with the first and second exons. Conveniently, the exon–intron boundaries conserve the canonical splice consensus sequences AG:*GT* and *CAG*:GC [26, 27]. According to this hypothesis, the predicted ORFs encode Hop proteins with the same number of amino acids than the other plant Hop members (Additional file 4: Figure S3 (C)). In addition, no significant similarity was found with a BLAST search using the 71 extra amino acids as query (not shown). Taken together, these results led us to the conclusion that the *Micromonas* sp. *hop* gene must enclose one intron located just after the first six codons (amino acids MADEHK). Thus, *Micromonas* sp. is classed in type B (Fig. 4), in which both non-collinear genes have a single intron, i.e., 1–1 (Table 1).

In type C, (*C. reinhardtii*), *hop* contains 12 introns while *fus* has 9. Contrary to the other members of division Chlorophyta, *C. reinhardtii* has accumulated a noticeable plethora of introns; some of them lie in positions shared with human and higher plants (See next section). In type D (*Physcomitrella patens*, a gymnosperm), each gene is located in a separate chromosome; *hop* comprises 7 introns and chl-*fus* 6. *Picea abies* − another gymnosperm−, belongs to type E, where *hop* has the same intron number as type D but the intron number is reduced to 3 in chl-*fus* gene. In type F (Musaceae (Monocot), Cucurbitaceae and 3 out of 5 Fabaceae (Dicot)) *hop* and chl-*fus* are not syntenic, but individual genes hold the same structure 6–3 of the greatest number of convergently transcribed genes in higher plants (type I). In type G, the exon–intron structure is the same of type I (6–3), but chl-*fus* was transposed to the 5′ flanking site of *hop*, and transcribed in the same direction (Fig. 4). In types H (5–3) and J (6–2), *hop* and chl-*fus* lack one intron, respectively, with regard to type I. It is concluded that during the evolutionary process, *hop* and chl-*fus* genes underwent extensive changes in their exon − intron structure, among unicellular photosynthetic eukaryotes, as well as in higher plants. It is interesting to notice that intron gain/loss affected both genes alike, by species. For example, *C. reinhardtii* (type C) *hop* and chl-*fus* conserved a plethora of introns (simultaneous intron gain?), while both genes in *O. lucimarinus* (type A) preserved only one (simultaneous intron loss?). This finding also applies to higher plants (Fig. 4).

### Intron position and phase as determinant of exon shuffling

In previous publications, it has been proposed that domain/module duplication has contributed to gene evolution through exon shuffling [28]. Bioinformatic analyses of vertebrate Hop orthologs suggested that TPR and DP domains behaved as a whole recombination unit due to the presence of phase-0 introns [6]. Phase-0 introns are the most favorable for exon duplication or shuffling without modifying the reading frame [28], and the human *hop* gene comprises TPR − DP modules surrounded by phase-0 introns. Likewise, by sequence alignments, it was hypothesized that EF-G emerged as a result of gene duplication/fusion events [29].

We analyzed the exon–intron topologies and intron phase distribution within plant *hop* and chl-*fus* genes, in order to reconstruct the molecular events leading to the emergence of present-day genes. As shown in Fig. 4, *hop* genes can be grouped in 6 classes of exon–intron structure (*h1*-*h6*), while *fus* genes are grouped in 5 classes (*f1*-*f5*). Considering only the *hop* gene, it contains zero, one or more introns in green algae. No introns were found either in *Ostreococcus lucimarinus* or *O. tauri* (Class *h1*), while *Micromonas* sp. was predicted to contain one 5′ phase-0 intron (Class *h2*). Contrary to the above mentioned Mamiellaceae family members (Fig. 4), *C. reinhardtii* (Chlamydomonadaceae) is the photosynthetic eukaryote with the greatest number of introns, with 12 short intragenic regions equally distributed within the coding region (Class *h3*). Although most of introns are phase-0 (9 out of 12), the recombinable module that most resembles those found in vertebrates is located between phase-0 introns I_*h*_ to VI_*h*_. This unit contains a complete TPR-DP-Ch. AA module, able to recombine by exon shuffling. The two gymnosperms, *P. patens* and *P. abies,* belong to Class *h4* with 7 introns located in equivalent positions. Class *h5* is the most abundant gene structure in higher plants (46 species). The first intron (I_*h*_, phase-0) splits the TPR2A domain. The rest of introns (3 out of 5 of phase-0) split the end of the TPR2A-coding exons and the C-terminal TPR2B − DP2-coding sequences. Finally, Class *h6* (*Aethionema arabicum*, one member out of 9 of the Brassicaceae family) exhibits the same exon–intron topology of Class *h5*, except that it lacks the Class *h5* intron V_*h*_, located within the DP2 domain (Fig. 4).

Disparities in intron number among *hop* orthologs were used to define classes *h1* to *h6* (Fig. 4). Additional file 5: Figure S4 shows that not all intron positions are conserved among higher plants. For example, the first intron (phase-0) *in C. reinhardtii hop* gene (I_*h*_), that locates between amino acids K and A (red line), is also found in *Micromonas* sp. but not in either *O. lucimarinus*, *L. alabamica* or *A. arabicum*. The second intron (phase-0) *in C. reinhardtii* (II_*h*_) locates between Y and A (blue line), and is exclusive to this species, and so forth. From Additional file 5: Figure S4 it is inferred that intron positions are mainly conserved among *hop* genes from higher plants, but only partially between higher plants and Chlorophyta or plants and human. For instance, *C. reinhardtii* introns II_*h*_ (0), III_*h*_ (0), IV_*h*_ (1), V_*h*_ (0), VI_*h*_ (0), VIII_*h*_ (2), IX_*h*_ (0) and XI_*h*_ (2) (blue lines) are exclusive to this green alga, while introns VII_*h*_ (0) and XII_*h*_ (0) (red lines) are shared with *L. alabamica* and *A. arabicum* and the rest of higher plants. Finally, higher plants contain introns restricted to Mono and Dicots, i.e., introns II_*h*_ (1), III_*h*_ (0) and IV_*h*_ (2) (red lines). Exceptionally, *A. arabicum* (Brassicaceae, Class *h5*) lacks the phase-0 intron V_*h*_ of higher plants (Class *h4*). In the bottom of Additional file 5: Figure S4 we represent the human Hop protein and its related introns. A careful comparison of intron location among plants and human reveals that human Hop shares two introns with *C. reinhardtii* (i.e., I_*h*_ (0) and X_*h*_ (0), red lines), but not with higher plants.

On the other hand, the chl-*fus* gene has undergone a higher reduction in intron number with respect to *hop*. The exon–intron structure was organized under five classes (*f1* to *f5*), according to the number and position of introns (Fig. 4). From algae to higher plants, the chl-*fus* gene contains a phase-1 intron that separates the signal peptide from the mature protein; this implies that a new exon coding for a N-terminal transit peptide was recruited, for the correct trafficking of cEF-G from cytoplasm to the plastids [30]. More precisely, Class *f1* embraces all predicted Mamiellaceae chl-*fus* genes with a single phase-1 intron, inserted between the chloroplast-targeting domain and the rest of the coding sequence (Fig. 4). On the contrary, the *C. reinhardtii* (Chlamydomonadaceae) chl-*fus* gene has eight additional phase-0 introns interspersed within the cEF-G coding region (Class *f2*). Class *f3* is a single form of chl-*fus* with five introns located in different places with respect to the rest of plant chl-*fus* genes. Class *f4* is the most prevalent exon–intron organization found in monocot and dicot plants (47 species). It contains two phase-0 introns, II_*f*_ and III_*f*_, apart from that coding for the transit peptide (phase-1), located within the 3′ half of the chl-*fus* gene (Fig. 4). Finally, only one member of Brassicaceae out of 9 (*Brassica rapa*) belongs to Class *f5*, which contains three exons and two introns. The *B. rapa* chl-*fus* gene lacks intron II_*f*_ with respect to Class *f4.*

### Molecular instability of the *hop* and chl-*fus* intergenic region

In several plant families, the intergenic region (IGR) between the *hop* and chl-*fus* genes suffered insertions and deletions. While 82 % of monocots and dicots preserve microcolinearity, the IGR among species is of variable length. For example, the shortest IGR belongs to *Leavenworthia alabamica* (188 bp), while the longest belongs to *Linum usitatissimum* (38523 bp). Nevertheless, the IGR region typically does not exceed 3500 bp (Additional file 1: Figure S1). IGR nucleotide sequences were analyzed by tBLASTn in order to identify potential ORFs. Plant retroviruses (or retrotransposons) and hypothetical genes were found in Monocots (*Elaeis guineensis* and *Phoenix dactylifera*) and Dicots (*Morus notabilis* and *Linum usitatissimum*), within IGRs >10 kb. For example, a putative pararetrovirus-like pseudogen was found within the 10 kb IGR of *M. notabilis*. In Additional file 6: Figure S5 (A), we show a ClustalW alignment between a putative polyprotein encoded by the *M. notabilis* IGR and a *Citrus endogenous* pararetrovirus, retrieved by BLAST (45 % identity). The *M. notabilis* predicted polyprotein is truncated by 12 aberrant stop codons, suggesting that it could be a pararetrovirus pseudogen. Furthermore, transposon-like repeated sequences were found in a number of species. For example, inverted repeat sequences of Miniature Inverted–Repeat Transposable Elements (MITEs) [31] were found within the IGR of *Oryza* spp (Additional file 6: Figure S5 (B)) and direct repeats of CACTA-like transposons [32] reside in *M. truncatula* IGR (not shown).

Two interesting cases of deletions within the IGR have been found in higher plants, which alter the 3′ untranslated region of the *hop* and chl-*fus* genes. In *Glycine max*, a plant with a predicted allopolyploidization event [33], two chl-*fus* genes were cloned and sequenced from cv. Ceresia (98 % identity between cEF-G1 and cEF-G2 proteins), both with *hop* genes in convergent transcription [8]. ClustalW alignments were performed between chl-*fus* genes of *G. max* cv. Ceresia and cDNAs from *G. max* cv. Williams, which contain three different poly-A sites (Additional file 7: Figure S6 (A)). An almost perfect match was found between the coding part and the 3′ untranslated region of the cDNAs, chl-*fus*1 and chl-*fus*2 genes; however, chl-*fus*1 drastically lacks identity 123 nucleotides downstream of the stop codon. A detailed nucleotide analysis allowed to conclude that a chromosomal deletion (ca. 680 bp) maps between the chl-*fus*1 and *hop*1 genes (Additional file 7: Figure S6 (B)).

A more severe case of IGR deletion is found in *A. thaliana*, in which the 3′ transcribed regions of the *hop* and chl-*fus* genes overlap. We show in Additional file 8: Figure S7 a chromosomal map of the *A. thaliana hop* and chl-*fus* genes, and three cDNAs of each gene, with multiple poly-A sites. As can be observed, the 3′ end of three *hop* and that of two chl-*fus* cDNAs overlap. Thus, in the strict sense, the IGR between *hop* and chl-*fus* genes is missing; nevertheless, according to the Genbank cDNA accessions, both genes are transcribed. We concluded that the IGR separating the *hop* and chl-*fus* genes in plants seems to be a target region for insertion and deletion (indel) events, making it genetically unstable.

## Discussion

### Microsynteny and coevolution of *hop* and chl-*fus* genes in plant genomes

In this paper, we provided extensive evidences unveiling the evolutionary changes suffered by the pair of plant *hop* and chl-*fus* genes, after the primary endosymbiotic events. One gene is typically of nuclear origin, while the other undoubtedly came from the precursors of modern chloroplasts; together, they could constitute an interesting model to draw conclusions on the genome rearrangement events during and after the transfer of chloroplast genes to the nucleus. The first remark is the outstanding conservation of microsynteny and microcolinearity, in spite of all genomic duplications, deletions, inversions, insertions, and translocation events that shape genomes [1]. Nevertheless, our results in Figs. 3 and 4 suggest that chl-*fus* was originally transferred from chloroplasts to a different chromosome from that of *hop* gene, in the proto-algal nuclear genome. This assumption is supported by the absence of microsynteny in green algae (prasinophytes) “which comprise the descendants of the primitive algae from which all green algal lineages, including the ancestors of land plants, evolved” [34, 35], and gymnosperms. Thus, the microcolinearity observed in mono and dicots should be the result of a recombination event, e.g., chromosome fusion, inversion or translocation [36], sometime before the appearance of angiosperms. A few monocot and dicot plant families also lack microsynteny, undoubtedly as a consequence of new genome rearrangements. While this issue rule out the possibility to discern details on the coevolution of nuclear vs. neighboring laterally transferred genes, each gene provides new insights to reconstruct the history of ancient nuclear genes.

A comparative analysis of the organization and expression patterns of divergent (←→) and convergent (→←) gene pairs was carried out for *Oryza sativa, Arabidopsis thaliana* and *Populus trichocarpa* [37] and citations included. The statistical analysis allowed to conclude that the “conservation of divergent or convergent arrangement among these species appears to be quite rare” excepting when “the divergent and convergent genes display strongly correlated expression levels —independently of the intergenic distance— or have one or more Gene Ontology (GO) classes in common”. The molecular significance of these findings relative to the pair of genes *hop* and chl-*fus* remains to be clarified, because no functional relationships between the Hop and cEF-G proteins, and/or common expression patterns have been so far reported.

The second interesting finding is the high degree of conservation of their encoded proteins, across evolution. Both genes arise from domain or module duplications [6, 28, 29, 38] but these events happened very early in time, before further intron gain and losses [39]. The phylogenetic trees in Figs. 1 and 2 reveal a high conservation of Hop and cEF-G proteins, in opposition to gene structure (Figs. 3 and 4) and DNA sequences (not shown), indicating that the conservation of their 1D to 3D protein structures are essential for their cellular functions. In all photosynthetic organisms under study, Hop keeps the typical domain structure of the fungi and animal orthologs (Additional file 5: Figure S4) [6]. This is an unexpected finding because in fungi, nematodes or insects, isoforms of the Hop protein lack DP1 or TPR1-DP1 domains [40], and it was assumed the existence of deletion mutants in plants. Therefore, the DP1-mutant found in *G. max* [6] is actually an exception rather than the rule. On the other hand, the cEF-G protein also remained virtually unchanged with respect to its prokaryotic ancestor (Fig. 1). Although plant cEF-G exhibits higher similarity with bacterial EF-G proteins, it shows a closer phylogenetic relationship with α-proteobacteria rather than with cyanobacteria, suggesting that the ancestor of cEF-G could be the α-proteobacterial progenitor of mitochondria [10]. Our results, based on the analysis of 53 plant species from 21 families, support that hypothesis without exception. Furthermore, it has been reported that two isoforms of EF-G have distinct roles in both translocation (EF-G1) and ribosome recycling (EF-G2) in a variety of species from bacteria [41] to mammals [42]. Phylogenetic trees built with a few of plant cEF-G sequences evidenced that cEF-G does not fall within one of these categories and forms a separate clade [10]; our phylogenetic analysis confirm this finding and reveals the existence of a single form of cEF-G proteins in photosynthetic organisms (Fig. 1). Thus, chloroplast protein synthesis translocation and ribosome recycling functions might be assumed by that unique form of cEF-G.

### Role of introns in *hop* gene evolution

The observed exon-intron structure of *hop* and chl-*fus* at different levels of organismal complexity (Figs. 3 and 4) leads to three main conclusions: First, several evidences support the hypothesis that both genes experienced intron gain and losses, before and after the transfer of chl-*fus* to the nuclear genome (Fig. 4). Second, whenever one gene gained (or lost) introns, the other one also did, suggesting a species-specific synchronized intron gain/loss: for example, in *Micromonas* sp. both non-collinear genes have a single intron, but in *C. reinhardtii* they gained multiple introns each [43, 44]. Last, exon shuffling played essential roles in the construction of these genes, making it feasible to reconstruct their evolutionary changes. Inexorably, recombination of symmetric exons/modules would keep the open reading frame uninterrupted by frameshifts [45–47].

It has been proposed that in vertebrates, the *hop* gene could have emerged from recombinable modules surrounded by introns of the same phase [6]. Our results provide new evidences that phase-0 introns were essential for *hop* gene construction in all eukaryotes. Based on the six gene topologies of Fig. 4 (*h1* to *h6*), we propose a model of the ancient events giving rise to the present-day structure of *hop* genes, with a minimum number of steps (see Additional file 9: Figure S8 and legend). Our model leads to some significant conclusions on the role of introns in *hop* gene evolution: i) Phase-0 introns were critical for serial exon shuffling recombinations of a primordial module [28, 47–49] composed of symmetric exons «miniexon – phase-0 intron – TPR domain − phase-0 intron − Ch. AA domain − phase-0 intron – DP domain», and giving rise to a ‘Proto-eukaryote *hop’*. Old phase-0 introns could be traced backward in time (i.e., green and purple, Additional file 9: Figure S8), a typical characteristic of ancient proteins constructed by shuffling of exon/modules [39, 48, 50, 51]. According to our evolutionary model, the human *hop* would preserve two old phase-0 introns as reminiscent of the original recombinable module. ii) The origin of introns is still a matter of debate [38, 52–54]. Nevertheless, it is difficult to explain the differences in intron number and position within *hop* genes, between animals and plants for example, or between *C. reinhardtii* and *Micromonas* sp., without considering a recent gain/loss of introns. According to our model, the gain/loss of introns by *hop* was a very dynamical process, leading to conclude that while some (phase-0) introns are very old, other (phase-0, 1 and 2) might be of recent origin, a long-standing hypothesis proposed for other eukaryotic genes e.g., the triose-phosphate isomerase gene [55]. Nevertheless, even though the gene was subjected to many recombinations, the ORF remained virtually unchanged (Fig. 2), except some shorter isoforms [6]. iii) It has been noticed a biased distribution of phase-0 introns immediately after the start codon in eukaryotic genes (vertebrates, invertebrates, fungi, plants, and protists), specially “at the boundaries of evolutionary modules in proteins without signal peptides and this effect is stronger in phylogenetically old proteins” [39, 56, 57]. Authors suggest that these introns should “allow the 5′ untranslated region (UTR) to participate in exon shuffling, so that different genes can exchange regulatory information” [57]. Interestingly, some of present-day *hop* genes exhibit a phase-0 intron downstream of the first 3 to 6 amino acids (Fig. 4 and Additional file 5: Figure S4). Since *hop* genes are regulated by different forms of stress [8, 58, 59], it is conceivable that it was by this way that the gene became stress-regulated. However, this well-disposed intron could also contribute to shuffle internal exons, specifically whole TPR or TPR-DP domains, a valid assumption in support of our evolutionary model (Additional file 9: Figure S8).

### Role of introns in chl-*fus* gene evolution

It has been proposed that the *fus* gene is actually a product of three consecutive duplication/fusion gene events [29]. Such kind of successive duplication/fusions of peptide segments becomes conceivable with the presence of phase-0 introns. However, since chloroplasts, and then *fus* genes are of prokaryotic origin, probably introns had not a significant role in the creation of the primordial *fus*, but some kind of illegitimate recombination [60]. Thus, present-day spliceosomal introns (all phase-0) were very likely gained after the transfer of chloroplast DNA to the nucleus [52]. Nevertheless, the phase-1 intron connecting the N-terminal transit peptide-coding exon and the mature protein may have played an important role in the functional establishment of chl-*fus* in the nucleus (Fig. 4) and its loss from the chloroplast. Certainly, experimental evidence supports the assumption that chloroplasts transfer genes to the nucleus at high frequencies. However, the rate of nuclear establishment is extremely low. This conclusion is supported by the low number of loci encoding transferred genes [13, 61–63]. Based on statistical analyses of Gene Ontology (GO) categories, functional enrichment analysis reveals that a large set of organelle related genes remained as single-copy genes, despite the species-specific polyploidization events that shaped angiosperm genomes [64, 65]. Some well-known chloroplast genes transferred to the nucleus of plant diploids like *rbcS* of *Arabidopsis thaliana* [66], and with polyploidization history like *Cab* of common wheat [67]) or *tuf* of soybean [68] constitute gene families, while a large quantity of nuclear genes that encode chloroplast proteins are present as a single copy per haploid set [69]. Interestingly, the chl-*fus* remained as a singleton in the whole 53 plant genomes studied here, even in those that have undergone polyploidization events. According to De Smeth et al., “it can be argued that single copy genes form a well-conserved core that is sensitive to either mutation or duplication”. Although the chl-*fus* gene effectively seems to be dosage sensitive, the reason of such singletons remains unknown, but two equally plausible hypotheses have been proposed [64].

Interestingly, all the 53 chl-*fus* genes under study contain intron I_*f*_ (Additional file 1: Figure S1). How this intron was acquired? A recent study on structural and sequence evolution in mitochondrial genes transferred to the nucleus revealed that the most frequent location of introns occurs within the noncore region (48 %), i.e., acquired sequences after gene transfer to the nucleus [70]. In only 8 % of genes, an intron lies between the core and noncore regions, “suggesting that the acquisition of the noncore region by exon shuffling is an uncommon mechanism” [70]. Although it is an infrequent case, the chl-*fus* genes acquired an intron between the core and noncore regions, and this intron has been strictly conserved across evolution from algae to angiosperms (Fig. 4). It may be inferred that intron I_*f*_ was the first intron gain of chl-*fus* and that this intron played a major role for the recruitment of a transit peptide and probably 5′ regulatory sequences, by exon shuffling [71].

Why phase-1 and not 0 or 2? A recent study on human secretory signal peptides revealed a biased distribution of phase-1 introns (49,9 %), in the vicinity of the signal peptide cleavage sites [72]. According to the authors “phase-1 introns most frequently split the four G↓GN codons encoding glycine”, that “are significantly enriched in positions −1, −3, −4 and −5”. Instead of this, for chl-*fus* genes, virtually all monocot and dicot phase-1 introns split codons G↓AU or G↓AC (Asp), and G↓AA or G↓AG (Glu), all fairly frequent split codons in all eukaryote taxonomic groups [73]. Exceptionally, *B. distachyon* (G↓GT) contains a triplet coding for the widespread Gly. Interestingly, this exception also applies for Chlorophyta: While *C. reinhardtii* keeps a G↓AC codon (Asp), *Micromonas*, *O. lucimarinus* and *O. tauri* contain G↓CN (Ala). Thus, it is tempting to speculate that the phase-1 intron that favored the fusion with the transit peptide-coding exon was originally splitting a G↓CN codon (Ala). Sometime in the evolution before the appearance of *C. reinhardtii*, G↓CN mutated to G↓AN (a C to A transversion).

### Would be compromised the integrity of the chl-*fus* gene for the future?

It has been elucidated that *hop* genes have a long history of gene rearrangements, which ended in the present-day form. These evidences support a natural susceptibility of the intergenic region to recombine: i) The chl-*fus* gene was recombined downstream of *hop* and this location might not have been a coincidence. ii) The IGR between *hop* and chl-*fus* has been in the midst of new chromosome rearrangements (e.g., gene inversion); such events must require some molecular propensity of that DNA to recombine. iii) We showed that in some plant species, retroviruses found suitable nucleotide sequences for transposition within the IGR. iv) Strikingly, in *G. max* the IGR almost disappeared, and in *A. thaliana*, it is totally absent. Thus, the unavoidable question is: where does that propensity to recombine come from? In our sequence analyses, we found a wide set of mobile elements inserted within the IGR of both monocots and dicots, indicating a high frequency of recombination. Interestingly, CACTA elements “frequently transduce host sequences” [32]; thus the presence of mobile DNA reinforces our assumption of a site of chromosomal instability. Currently, there is no database available for an extensive search of recombination “hot spots” [74], covering all the plant species studied here. However the possibility that chl-*fus* and *hop* genes are in the middle of a recombination “hot spot” should not be discarded. Regardless of the basis of such DNA instability, one may assume that the propensity to gain or loss nucleotides has come to affect the integrity of 3′ flanking sequences. Since there are no other genetic loci coding for the cEF-G protein (contrary to *hop* gene families), there would be a real risk of having plant mutants lacking the whole or part of the chl-*fus* genes. Actually, it may already have happened a number of times but such mutants could be unviable, in theory. Paradoxically, the chl-*fus* gene was transposed into a point of DNA instability and heretofore it continues to occupy the same and unique locus in the plant genome, judging by the high conserved microsynteny.

## Conclusions

In this study, we performed a deep analysis of the structure of two convergently transcribed nuclear genes, *hop* (nuclear origin) and chl-*fus* (plastid origin). We concluded that their convergence was a product of chromosome recombination rather than direct transfer of chl-*fus* from the chloroplast, downstream of *hop*. The exon–intron organization and intron phase of both genes agree with exon shuffling events, giving rise to exon/module duplications and transit peptide recruiting for chloroplast protein import. We showed evidences of instability of the intergenic region and susceptibility to recombination, that could favored the recombination of chl-*fus* within this region. Finally, the pair of genes *hop* and chl-*fus* should be useful as genetic markers, on the basis of microcolinearity in higher plants but not in Chlorophyta.

## Methods

### Accession numbers and exon assembly

The *Glycine max* chl-*fus* gene [GenBank: X71439] [12] was used as a query sequence for BLAST searches in Genbank [75]. *Picea abies* contigs were retrieved by BLAST from Dendrome Project, http://dendrome.ucdavis.edu. Accession numbers of retrieved contigs are in Table 1. Exon assembly was resolved using Geneious software [76] combined with manual adjustments. *G. max* cEF-G [12] and human Hop [58] were used as reference for exon assembly and protein domain definition. cDNAs from *A. thaliana* (cv. Columbia) were: cDNA1 [GenBank:BX815512], cDNA2 [GenBank:AK228637] and cDNA3 [GenBank:NM_104952] for *hop* gene and cDNA1 [GenBank:NM_104951], cDNA2 [GenBank:AK221774] and cDNA3 [GenBank:AY142646] for chl-*fus* gene. Sequence alignments were performed using ClustalW (EBI) under default parameters [77].

### Intron phase definition

Intron phase was assigned as stated by Patty [48]. Phase-0 introns split the open reading frame (ORF) within two codons, e.g., 5′GGC **CAG**:GT— intron— AG:**GTC** ACG3′. Phase-1 introns split the ORF between the first and second nucleotides of a codon, e.g., 5′CCA **G**:GT—intron—AG:**GT** CAC3′. Phase-2 introns interrupt the ORF between the second and third nucleotides of a codon, e.g., 5′GGC **AG**:GT—intron—AG:**G** TCA3′. Recombinable modules are defined as a set of exons flanked by introns of the same phase, typically phase-0 [6].

### Phylogenetic analysis

Maximum Likelihood phylogenetic trees were constructed using RaxML program version 7.3.0 [78]. All other settings were left as default, with 1000 replicates for bootstrapping. Human Hop protein [GenBank:NP_006810] and *A. thaliana* mEF-G [GenBank:NC_003070] were used as outgroups. Additional EF-G sequences were: *A. caulinodans* ORS 571 [GenBank:YP_001525473], *A. fabrum* str. C58 [GenBank:NP_354925], *F. alni* ACN14a [GenBank:YP_711337], *K. radiotolerans* [GenBank: SRS30216YP_001360437], *R. prowazekii* str. Madrid E [GenBank: NP_220524], *Synechococcus* sp. [GenBank: P18667] and *S. coelicolor* [GenBank: NP_628821].

### Hydrophobic cluster analysis (HCA)

Through the HCA method [79], we circumscribed the TPR and DP domain limits of orthologous Hop proteins. Besides, protein alignments were performed by this method. HCA is a method of protein analysis, implying the representation of amino acid sequences into a 2D space. The image is duplicated to exhibit the neighboring residues for each amino acid. Hydrophobic amino acids form clusters that correspond to the centers of regular secondary structures [80]. The shapes of the clusters are a keen indication of the nature of the secondary structure [81]. Clusters are roughly vertical when they code for a strand, while helixes are fairly horizontal. In a 2D protein alignment, the conserved shapes of the clusters are more important than the exact conservation of the residues inside the clusters. Thus, HCA allows alignments between very distantly related proteins, with as low as 10 % identity. Additional sequences used in HCA alignments were: *O. lucimarinus* [GenBank:NC_009360], *Micromonas* sp. RCC299 [GenBank:NC_013040], *C. reinhardtii* [GenBank:NW_001843572], *L. alabamica* [GenBank:ASXC01000179], *A. arabicum* [GenBank:ASZG1007785] and human [GenBank:NC_000011].

## Additional files

Additional file 1: Figure S1. Detailed gene structure and chromosomal arrangement of the pair of genes *hop* and chl-*fus*, for the 53 plant genomes under study. CO: classification by microcolinearity (categories I to III); GA: classification by gene arrangement, according to the exon–intron structure of both combined *hop* and chl-*fus* (categories A to J). Arabic and roman numbers represent intron phase (0, 1, or 2) and succession of introns from I to I + n, respectively; *hop* introns are named as I_*h*_, II_*h*_, III_*h*_, etc., and chl-*fus* introns are named as I_*f*_, II_*f*_, III_*f*_, etc. Exons coding for TPR and DP domains are color-coded according to conventions of Figs. 3 and 4. Non-syntenic genes are drawn on separate chromosomes. (PDF 815 kb) Additional file 2: Table S1. Plant species whose *hop* and chl-*fus* genes do not locate on the same chromosome. N.A., Not Available. (PDF 390 kb) Additional file 3: Figure S2. Graphic representation of microsynteny between *hop* and chl-*fus* genes among all plant species studied. (A) Plant species are ranked in the taxonomic order Chlorophyta, Gymnosperms (Gymnos); Angiosperms (Angios): Monocots (M) and Dicots. (B) Plant species are ranked by microsyntenic categories I, II and III. Arrows represent the transcriptional orientation of *hop* and chl-*fus* genes: An arrow (→), *hop* gene. An arrow with dot at the opposite end, chl-*fus* gene. (PDF 263 kb) Additional file 4: Figure S3. Prediction of an intron (dotted vertical line) in *Micromonas* sp. *hop* gene (GenBank: XP_002500383), downstream of the first six codons. (A) HCA alignment of the N-terminal amino acids of *Micromonas* sp. and *A. thaliana* Hop proteins. Extra 71 amino acids in the *Micromonas* sp. Hop protein are bordered by a rounded rectangle. Vertical lines connect analogous positions in both proteins. Conserved hydrophobic clusters are gray shaded. Relevant nonhydrophobic identities are indicated by circles on black background. The way to read the sequence and secondary structures, as well as special symbols, are indicated in the inset. (B) Predicted translation of the 5′ regions for *Micromonas* sp. and *C. reinhardtii hop* genes. We propose that nucleotides in bold belong to a phase-0 intron, which is in frame with the first and second exons. Splice sites are in italic and underlined. (C) ClustalW alignment [77] of the N-terminal amino acids of predicted *Micromonas* sp., *C. reinhardtii* and *A. thaliana* Hop proteins. The arrow indicates the position of the putative intron I_*h*_ in *Micromonas* sp., and *C. reinhardtii hop* genes. (PDF 595 kb) Additional file 5: Figure S4. 2D-alignment of plant Hop proteins from members of five categories of exon–intron organization of *hop* genes (*h1* to *h6*). The way to read the sequence and special symbols is the same of Additional file 4: Figure S3 (A). Solid vertical colored lines mark intron positions and dashed lines connect equivalent sites in orthologous proteins. Blue introns: species-specific introns; red introns: Introns shared among classes *h1* to *h6*; Human Hop protein is represented at the bottom. Yellow introns: Human-specific introns. Gray boxes: strict identities with respect to the *A. alabamica* VPEVEKKLEPEPEP triplet repeat (yellow box). Roman and Arabic numbers represent the succession of introns from I to I + n and intron phase (0, 1, or 2), respectively. TPR, DP and Ch. AA domains are bordered by rectangles with rounded corners. Domain names are on the top. (PDF 2687 kb) Additional file 6: Figure S5. Hypothetical genes found within the IGR between the *hop* and chl-*fus* genes. (A) ClustalW alignment between an inferred pseudogen encoded by the *Citrus endogenous* (Ce) pararetrovirus (Genbank:KF800044) and the *M. notabilis* IGR (Mn), found in this work. Colored boxes represent signature domains, including the viral movement protein, zinc finger, reverse transcriptase, and RNAse H. (*): internal stop codons. (B) Three predicted secondary structures [82, 83] of the inverted repeat sequences of a Miniature Inverted–Repeat Transposable Element (MITE) found within the IGR of *Oryza* spp; dG: Gibbs free energy. (PDF 689 kb) Additional file 7: Figure S6. The IGR between the *hop*1 and chl-*fus*1 genes of *G. max* cv. Ceresia is shorter than that of *hop*2 and chl-*fus*2. (A) Multalin multiple alignment of the 3′ region of *G. max* cv. Ceresia chl-*fus*1 and chl-*fus*2 genes with three *G. max* cv. Williams cDNAs. Translational termination stop codons (TAA) are bold and underlined (red arrow). Blue nucleotides in chl-*fus*1 and chl-*fus*2 genes: Mismatched positions with respect to cDNAs. Identity between chl-*fus*1 and chl-*fus*2 + cDNA sequences stop 123 positions downstream of the stop codon (blue arrow). A (n): poly-A tail. (B) Structure of the two genetic loci consisting each of a pair of *hop* and chl-*fus* genes, in *G. max* cv. Ceresia. Note that *hop* and chl-*fus* genes keep opposite polarity. Vertical arrows indicate deleted nucleotides (ca. 680 bp) in chl-*fus*1. Intron number and phase are the same of Fig. 4. (PDF 515 kb) Additional file 8: Figure S7. In *A. thaliana*, the *hop* and chl-*fus* genes overlap in the 3′ end*.* (A) Graphic view of the IGR separating the *hop* and chl-*fus* genes in *A. thaliana*. Last exons and 3′ non-coding ends are color-coded: red, *hop* gene; blue, chl-*fus* gene. The long horizontal arrows represent retrieved cDNAs from Genbank (see Methods for accession numbers). The shaded box covers the overlapping 3′ non-coding cDNA ends. (A) n: poly-A tails. (B) Topology of the *hop* and chl-*fus* genes, showing the absence of IGR region and overlapping 3′ ends. (PDF 354 kb) Additional file 9: Figure S8. Hypothetical evolutionary model of the *hop* gene. (A) Inside the nucleus of the primitive eukaryote, successive recombinations of a primary «mini-exon − phase-0 intron – TPR domain − phase-0 intron − Ch. AA − phase-0 intron – DP domain» module led to the formation of a ‘proto-eukaryote *hop* gene’. Gray, pink and yellow boxes enclose remaining exons and introns. Through the modular assembly of the ‘proto-eukaryote *hop*’, two phase-0 introns remained (one green, one purple) (B) Evolution from the ‘proto-eukaryote form’ to the present-day human *hop* gene. The green and purple phase-0 introns were preserved. Furthermore, eleven new introns were gained in the process. (C) The ‘proto-eukaryote form’ evolved to ‘pre-plant form’. The purple intron was lost, leading to the fusion of the DP1 and TPR2A domains; meanwhile, the blue and red introns were gained. (D) The ‘pre-plant form’ gradually reduced its intron number to zero, giving rise to contemporary *Micromonas* sp. and *O. lucimarinus hop* genes. (E) Nevertheless, on the way to the evolution towards more complex photosynthetic eukaryotes, the ‘pre-plant form’ eventually acquired a broad number of new introns such as in *C. reinhardtii,* gymnosperms and angiosperms (e.g., *A. thaliana*), but conserving the blue and red introns. (PDF 177 kb)

## Abbreviations

Bp: base pairs
Dp domain: a domain rich in Asp (D) and Pro (P) repeats
cEF-G: chloroplast-specific translation elongation factor G
mEF-G: mitochondrial-specific translation elongation factor G
Hop: Heat shock protein (HSP) organizing protein
cTP: Chloroplast Transit Peptide
chl-*fus*: gene encoding the cEF-G
TPR: Tetratricopeptide repeat
*hop*: gene encoding the Hop protein
ORF: Open Reading Frame
Ch. AA: Charged amino acids
