# Supplementary material for: Recombination of chl-fus gene (Plastid Origin) downstream of hop: a locus of chromosomal instability
Source: BMC Genomics. 2015 Aug 4;16(1):573. doi: 10.1186/s12864-015-1780-1 (PMC4522979; doi:10.1186/s12864-015-1780-1)
Supplement: Additional file 1: Figure S1. — Detailed gene structure and chromosomal arrangement of the pair of genes hop and chl-fus, for the 53 plant genomes under study. CO: classification by microcolinearity (categories I to III); GA: classification by gene arrangement, according to the exon–intron structure of both combined hop and chl-fus (categories A to J). Arabic and roman numbers represent intron phase (0, 1, or 2) and succession of introns from I to I + n, respectively; hop introns are named as Ih, IIh, IIIh, etc., and chl-fus introns are named as If, IIf, IIIf, etc. Exons coding for TPR and DP domains are color-coded according to conventions of Figs. 3 and 4. Non-syntenic genes are drawn on separate chromosomes. (PDF 815 kb) [file 12864_2015_1780_MOESM1_ESM.pdf]

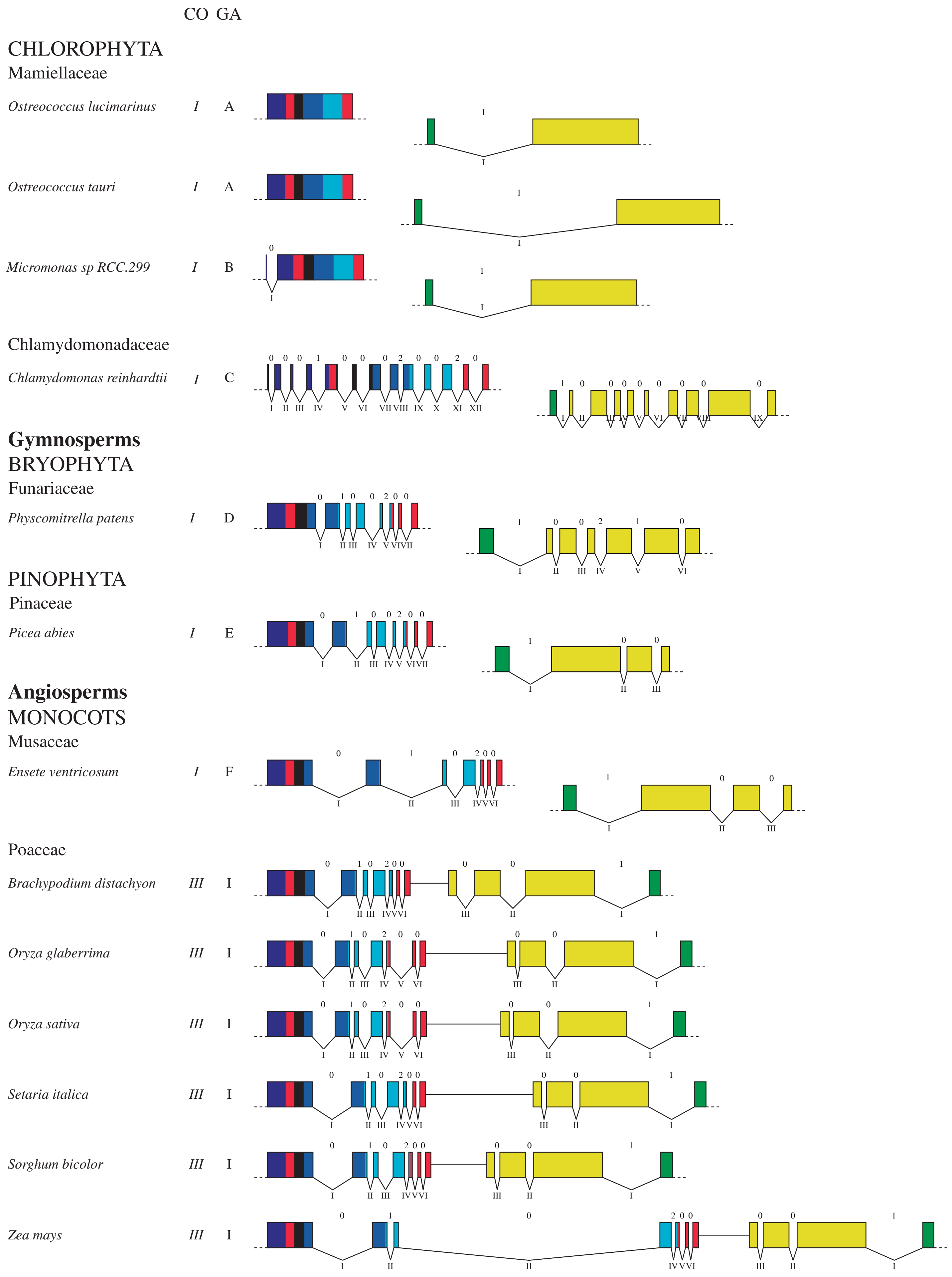
CO GA
CHLOROPHYTA
Mamiellaceae
Ostreococcus lucimarinus I A
Ostreococcus tauri I A
Micromonas sp RCC.299 I B
Chlamydomonadaceae
Chlamydomonas reinhardtii I C
Gymnosperms
BRYOPHYTA
Funariaceae
Physcomitrella patens I D
PINOPHYTA
Pinaceae
Picea abies I E
Angiosperms
MONOCOTS
Musaceae
Ensete ventricosum I F
Poaceae
Brachypodium distachyon III I
Oryza glaberrima III I
Oryza sativa III I
Setaria italica III I
Sorghum bicolor III I
Zea mays III I

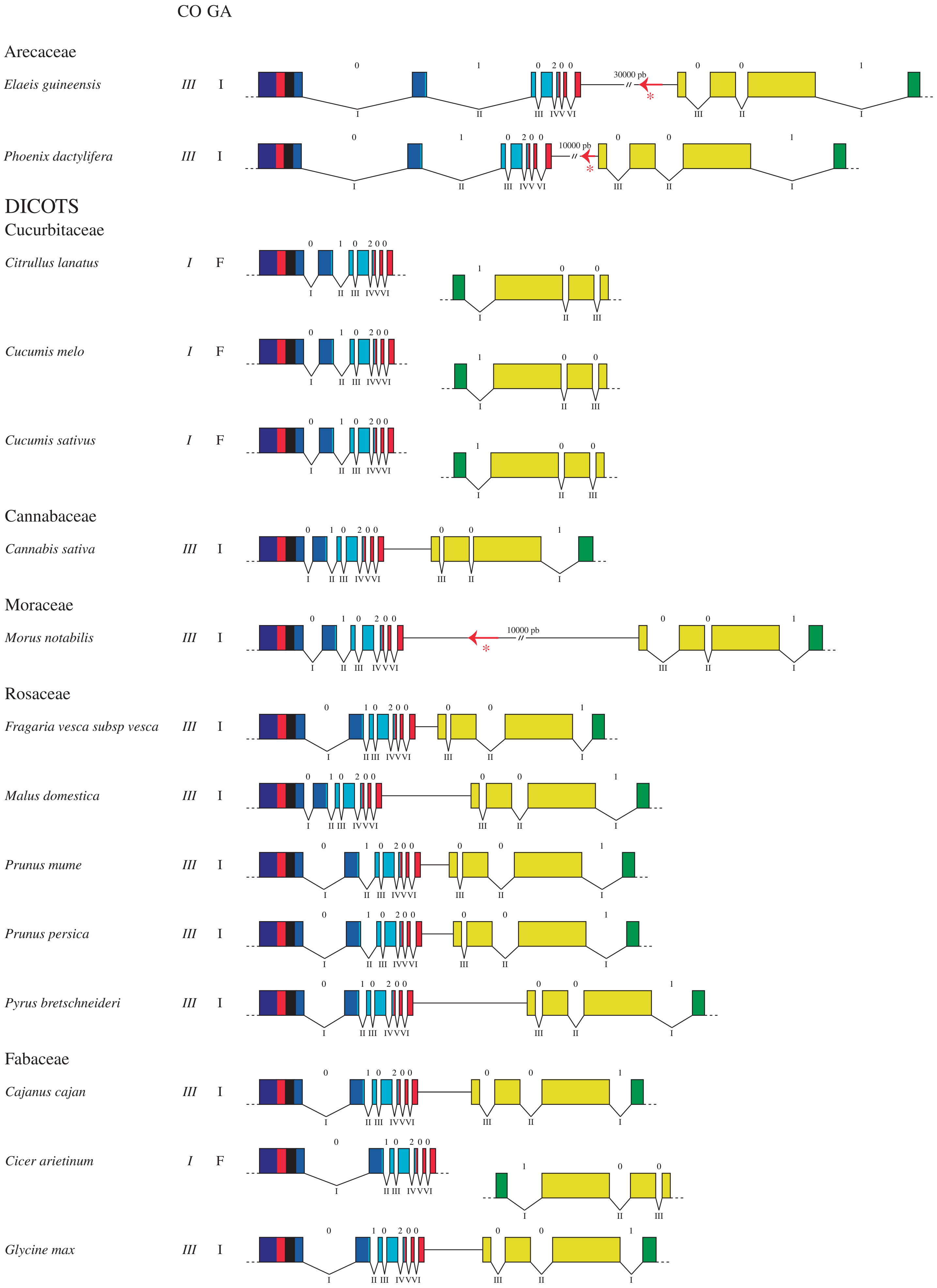
CO GA
Arecaceae
Elaeis guineensis III I
30000 pb
Phoenix dactylifera III I
10000 pb
DICOTS
Cucurbitaceae
Citrullus lanatus I F
Cucumis melo I F
Cucumis sativus I F
Cannabaceae
Cannabis sativa III I
Moraceae
Morus notabilis III I
10000 pb
Rosaceae
Fragaria vesca subsp vesca III I
Malus domestica III I
Prunus mume III I
Prunus persica III I
Pyrus bretschneideri III I
Fabaceae
Cajanus cajan III I
Cicer arietinum I F
Glycine max III I

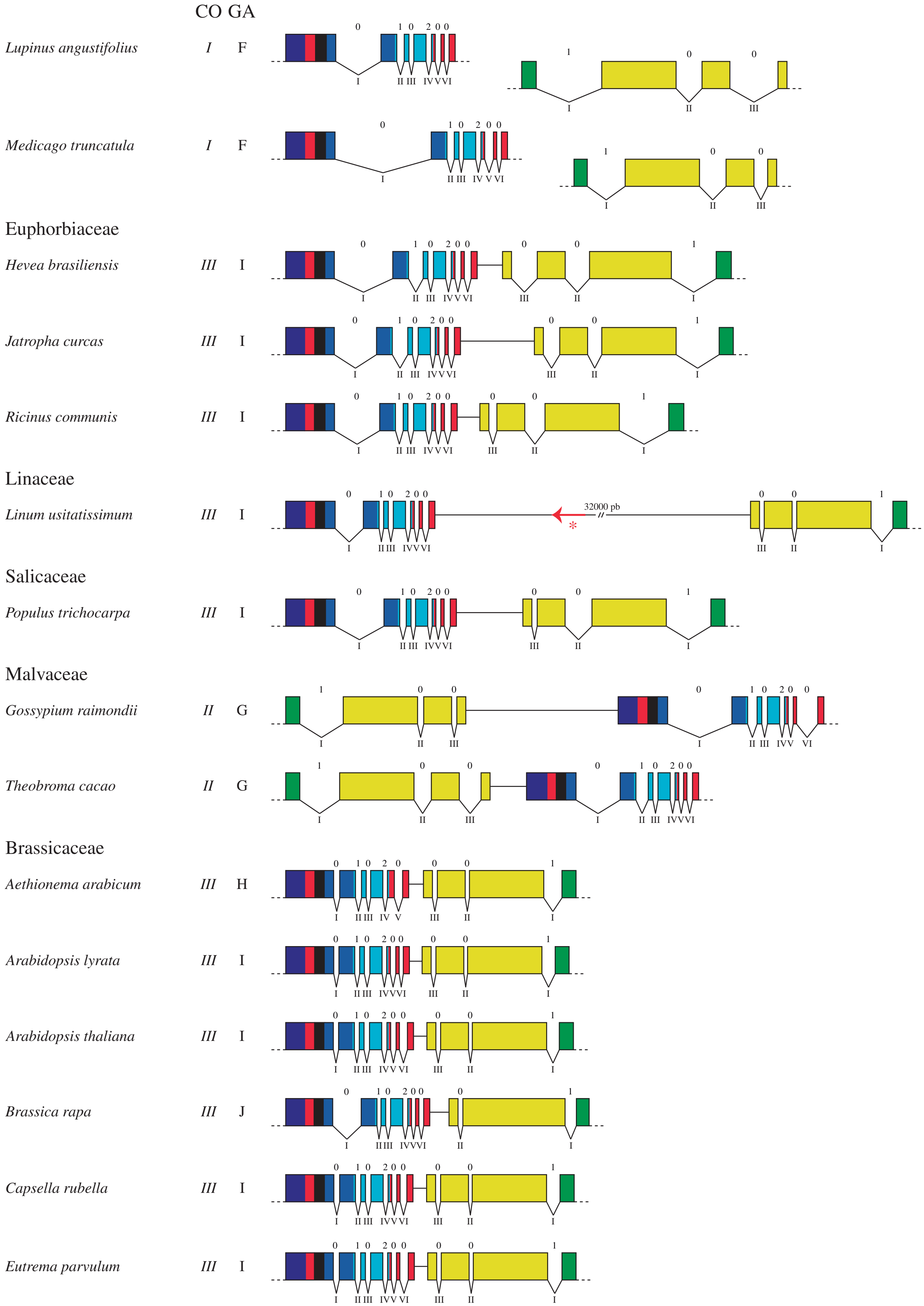
CO GA
Lupinus angustifolius I F
Medicago truncatula I F
Euphorbiaceae
Hevea brasiliensis III I
Jatropha curcas III I
Ricinus communis III I
Linaceae
Linum usitatissimum III I
32000 pb
*
Salicaceae
Populus trichocarpa III I
Malvaceae
Gossypium raimondii II G
Theobroma cacao II G
Brassicaceae
Aethionema arabicum III H
Arabidopsis lyrata III I
Arabidopsis thaliana III I
Brassica rapa III J
Capsella rubella III I
Eutrema parvulum III I

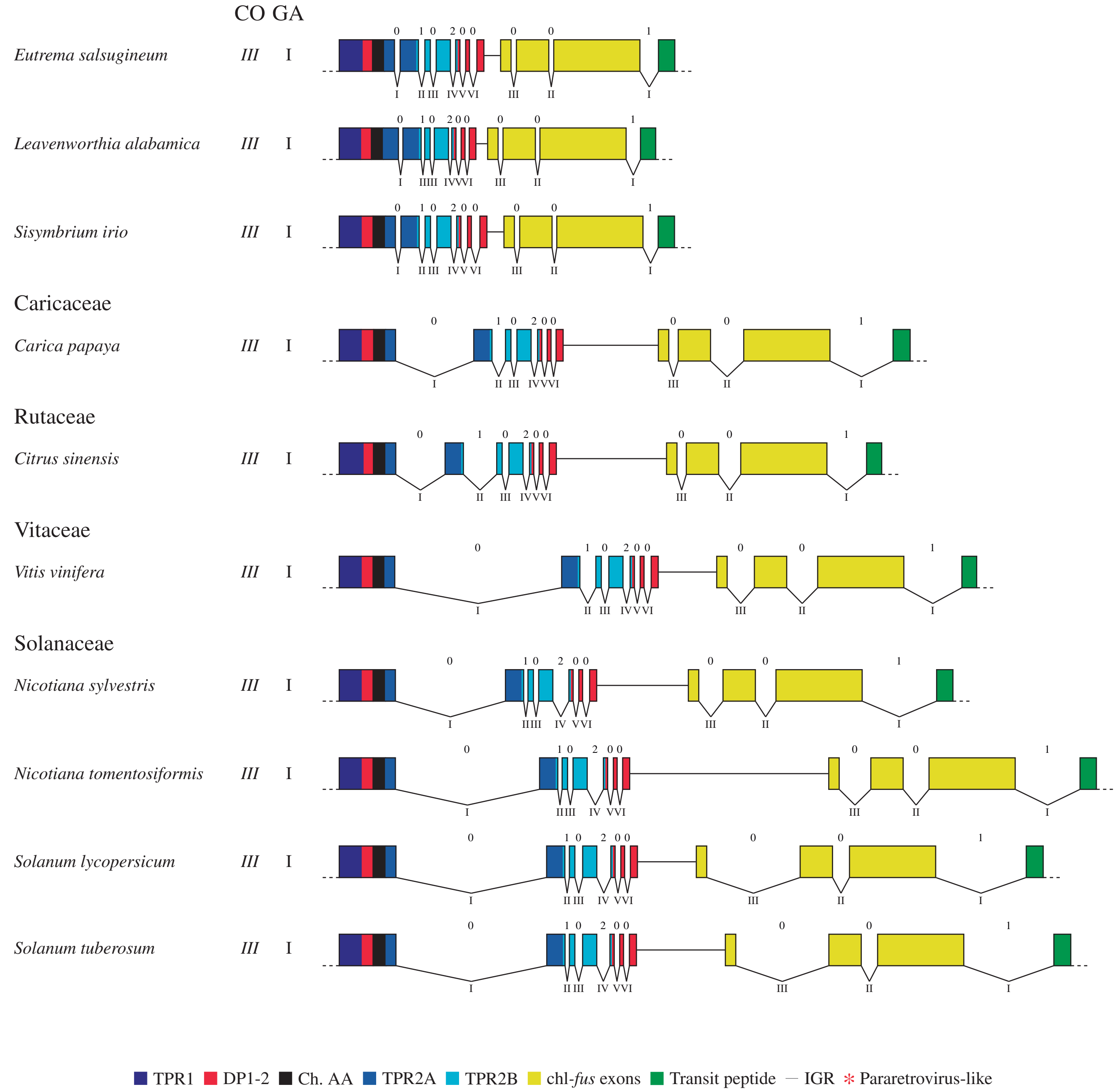
CO GA
Eutrema salsugineum III I
Leavenworthia alabamica III I
Sisymbrium irio III I
Caricaceae
Carica papaya III I
Rutaceae
Citrus sinensis III I
Vitaceae
Vitis vinifera III I
Solanaceae
Nicotiana sylvestris III I
Nicotiana tomentosiformis III I
Solanum lycopersicum III I
Solanum tuberosum III I
TPR1
DP1-2
Ch. AA
TPR2A
TPR2B
chl-fus exons
Transit peptide
IGR
Pararetrovirus-like
