# Supplementary material for: Recombination of chl-fus gene (Plastid Origin) downstream of hop: a locus of chromosomal instability
Source: BMC Genomics. 2015 Aug 4;16(1):573. doi: 10.1186/s12864-015-1780-1 (PMC4522979; doi:10.1186/s12864-015-1780-1)
Supplement: Additional file 2: Table S1. — Plant species whose hop and chl-fus genes do not locate on the same chromosome. N.A., Not Available. (PDF 390 kb) [file 12864_2015_1780_MOESM2_ESM.pdf]

Species	Family	Chl-*fus* Accession Number	Chromosome number	*Hop* Accession Number	Chromosome number
Micromonas sp. RCC299	Mamiellaceae	XP_002500081	2	XP_002500383	3
Ostreococcus lucimarinus	Mamiellaceae	XP_001419031	7	XP_001418158	6
Ostreococcus tauri	Mamiellaceae	XM_003080500	7	XM_003079642	6
Chlamydomonas reinhardtii	Chlamydomonadaceae	XM_001701793	N.A.	XP_001691869	N.A.
Ensete ventricosum	Musaceae	AMZH01015354	N.A.	AMZH01008475	N.A.
Physcomitrella patens	Funariaceae	NW_001865607	N.A.	XP_001784483	N.A.
Picea abis	Pinaceae	MA_10426940	8	MA_10431292	7
Citrullus lanatus	Cucurbitaceae	AGCB01004585	N.A.	AGCB01006484	N.A.
Cucumis melo	Cucurbitaceae	CAJI01003926	N.A.	CAJI01012439	N.A.
Cucumis sativus	Cucurbitaceae	XM_004147564	N.A.	XM_004147890	N.A.
Cicer arietinum	Fabaceae	XM_004515686	8	XM_00451602	N.A.
Lupinus angustifolius	Fabaceae	AOCW01054016	N.A.	AOCW01121688	N.A.
Medicago truncatula	Fabaceae	NC_016410	4	NC_016411	5
