# Supplementary figures and images for: Recombination of chl-fus gene (Plastid Origin) downstream of hop: a locus of chromosomal instability
Source: BMC Genomics. 2015 Aug 4;16(1):573. doi: 10.1186/s12864-015-1780-1 (PMC4522979; doi:10.1186/s12864-015-1780-1)

A) By taxonomy

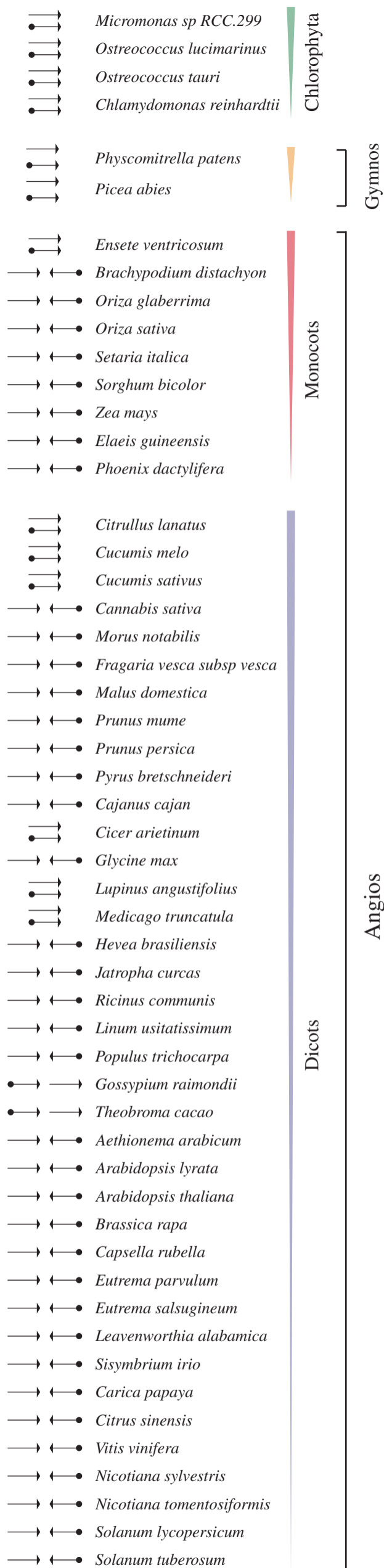

**B) By type of genome arrangement**

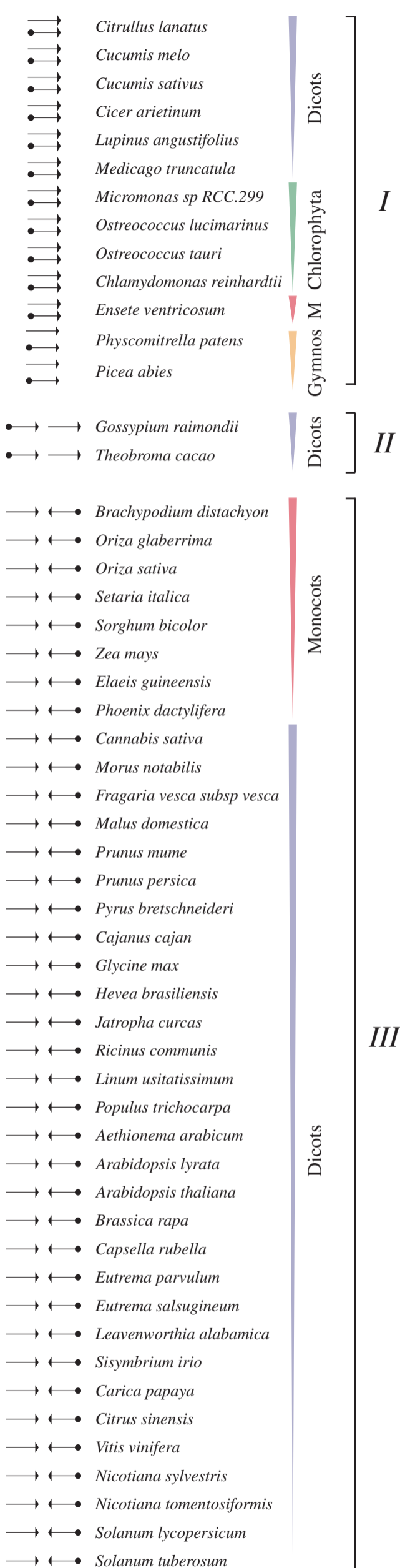

Supplement: Additional file 3: Figure S2. — Graphic representation of microsynteny between hop and chl-fus genes among all plant species studied. (A) Plant species are ranked in the taxonomic order Chlorophyta, Gymnosperms (Gymnos); Angiosperms (Angios): Monocots (M) and Dicots. (B) Plant species are ranked by microsyntenic categories I, II and III. Arrows represent the transcriptional orientation of hop and chl-fus genes: An arrow (→), hop gene. An arrow with dot at the opposite end, chl-fus gene. (PDF 263 kb) [file 12864_2015_1780_MOESM3_ESM.pdf]

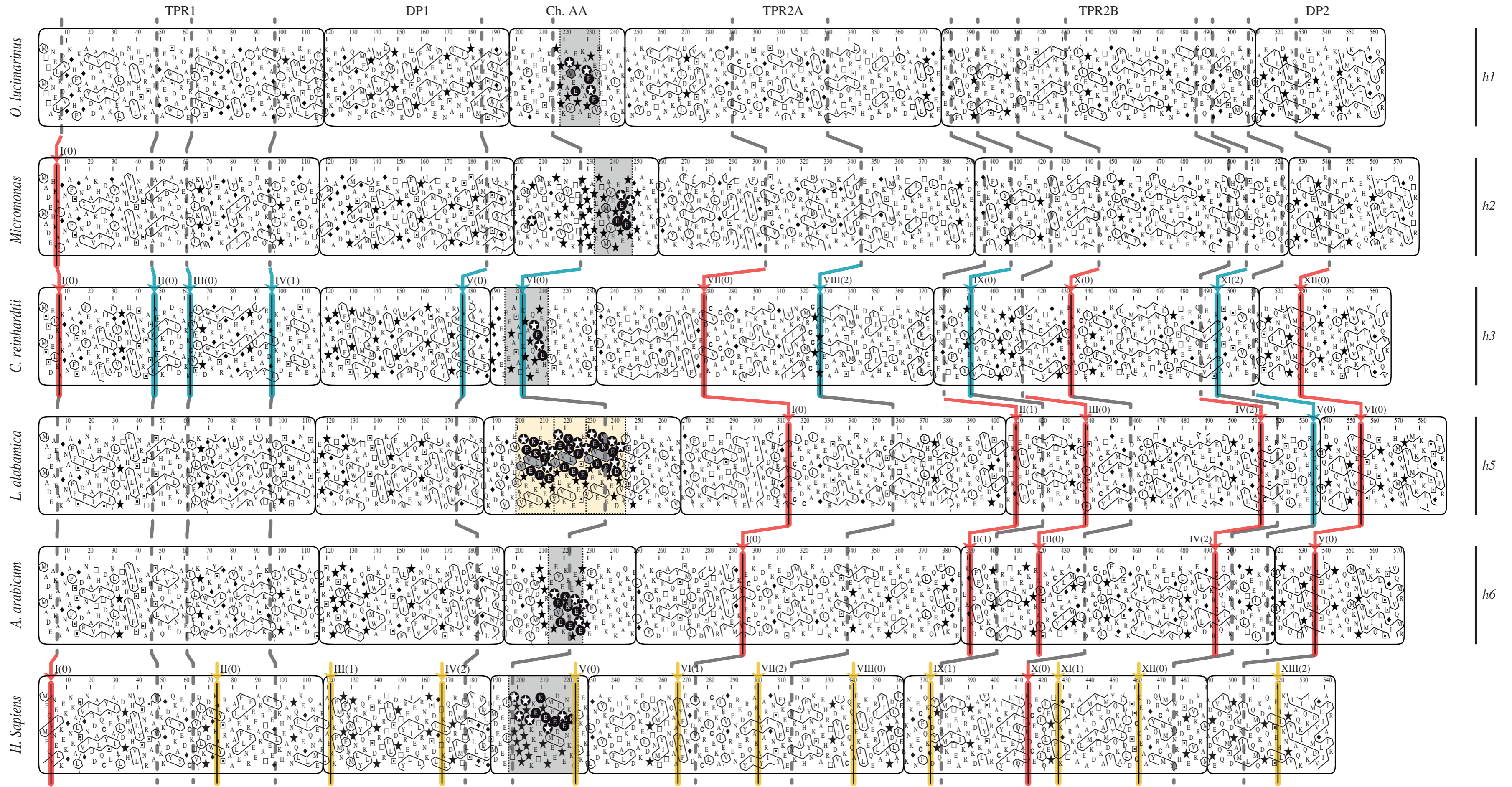

Supplement: Additional file 5: Figure S4. — 2D-alignment of plant Hop proteins from members of five categories of exon–intron organization of hop genes (h1 to h6). The way to read the sequence and special symbols is the same of Additional file 4: Figure S3 (A). Solid vertical colored lines mark intron positions and dashed lines connect equivalent sites in orthologous proteins. Blue introns: species-specific introns; red introns: Introns shared among classes h1 to h6; Human Hop protein is represented at the bottom. Yellow introns: Human-specific introns. Gray boxes: strict identities with respect to the A. alabamica VPEVEKKLEPEPEP triplet repeat (yellow box). Roman and Arabic numbers represent the succession of introns from I to I + n and intron phase (0, 1, or 2), respectively. TPR, DP and Ch. AA domains are bordered by rectangles with rounded corners. Domain names are on the top. (PDF 2687 kb) [file 12864_2015_1780_MOESM5_ESM.pdf]

[illegible]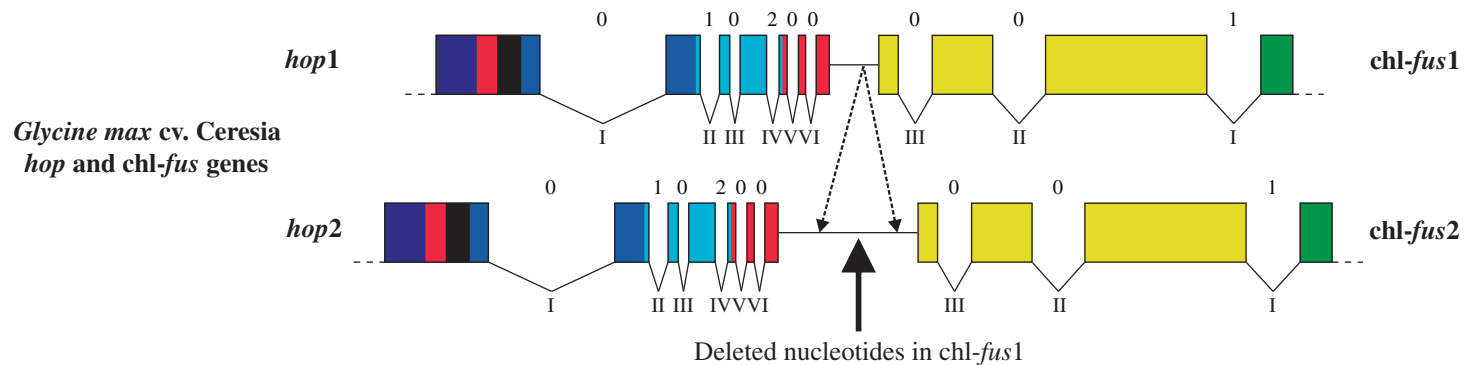

Supplement: Additional file 7: Figure S6. — The IGR between the hop1 and chl-fus1 genes of G. max cv. Ceresia is shorter than that of hop2 and chl-fus2. (A) Multalin multiple alignment of the 3′ region of G. max cv. Ceresia chl-fus1 and chl-fus2 genes with three G. max cv. Williams cDNAs. Translational termination stop codons (TAA) are bold and underlined (red arrow). Blue nucleotides in chl-fus1 and chl-fus2 genes: Mismatched positions with respect to cDNAs. Identity between chl-fus1 and chl-fus2 + cDNA sequences stop 123 positions downstream of the stop codon (blue arrow). A (n): poly-A tail. (B) Structure of the two genetic loci consisting each of a pair of hop and chl-fus genes, in G. max cv. Ceresia. Note that hop and chl-fus genes keep opposite polarity. Vertical arrows indicate deleted nucleotides (ca. 680 bp) in chl-fus1. Intron number and phase are the same of Fig. 4. (PDF 515 kb) [file 12864_2015_1780_MOESM7_ESM.pdf]

A)

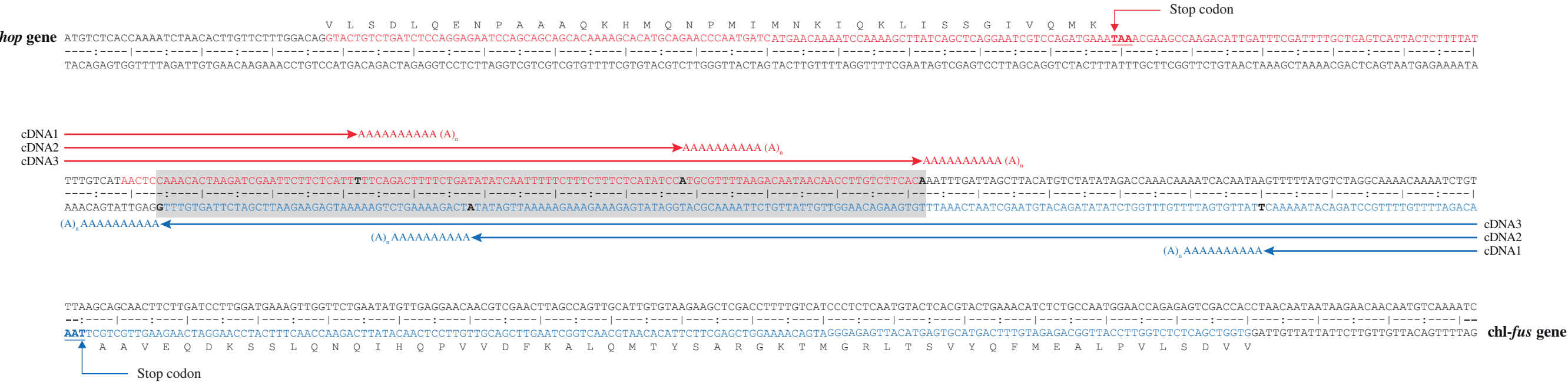

B)

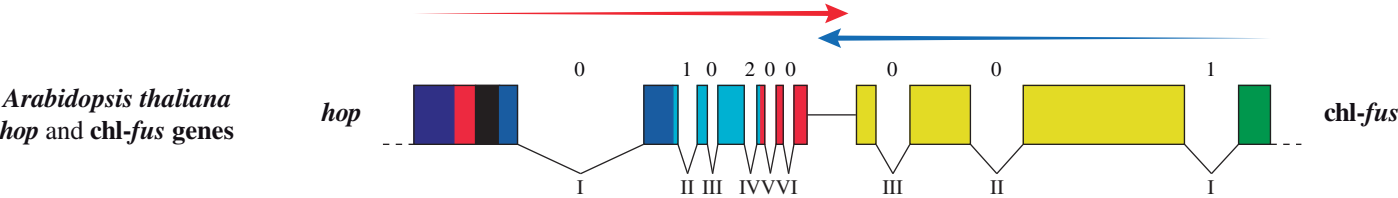

Supplement: Additional file 8: Figure S7. — In A. thaliana, the hop and chl-fus genes overlap in the 3′ end. (A) Graphic view of the IGR separating the hop and chl-fus genes in A. thaliana. Last exons and 3′ non-coding ends are color-coded: red, hop gene; blue, chl-fus gene. The long horizontal arrows represent retrieved cDNAs from Genbank (see Methods for accession numbers). The shaded box covers the overlapping 3′ non-coding cDNA ends. (A) n: poly-A tails. (B) Topology of the hop and chl-fus genes, showing the absence of IGR region and overlapping 3′ ends. (PDF 354 kb) [file 12864_2015_1780_MOESM8_ESM.pdf]

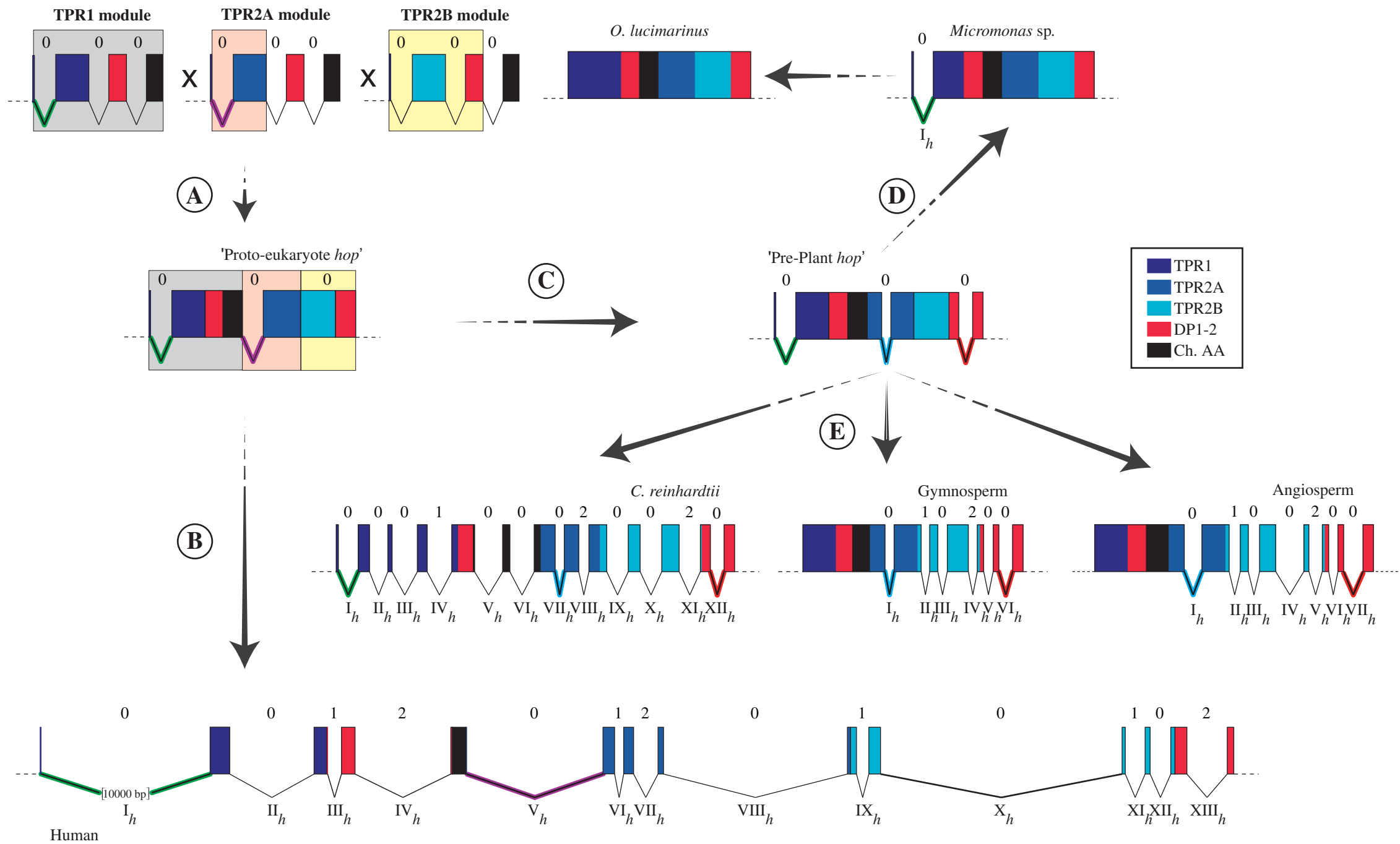

Supplement: Additional file 9: Figure S8. — Hypothetical evolutionary model of the hop gene. (A) Inside the nucleus of the primitive eukaryote, successive recombinations of a primary «mini-exon − phase-0 intron – TPR domain − phase-0 intron − Ch. AA − phase-0 intron – DP domain» module led to the formation of a ‘proto-eukaryote hop gene’. Gray, pink and yellow boxes enclose remaining exons and introns. Through the modular assembly of the ‘proto-eukaryote hop’, two phase-0 introns remained (one green, one purple) (B) Evolution from the ‘proto-eukaryote form’ to the present-day human hop gene. The green and purple phase-0 introns were preserved. Furthermore, eleven new introns were gained in the process. (C) The ‘proto-eukaryote form’ evolved to ‘pre-plant form’. The purple intron was lost, leading to the fusion of the DP1 and TPR2A domains; meanwhile, the blue and red introns were gained. (D) The ‘pre-plant form’ gradually reduced its intron number to zero, giving rise to contemporary Micromonas sp. and O. lucimarinus hop genes. (E) Nevertheless, on the way to the evolution towards more complex photosynthetic eukaryotes, the ‘pre-plant form’ eventually acquired a broad number of new introns such as in C. reinhardtii, gymnosperms and angiosperms (e.g., A. thaliana), but conserving the blue and red introns. (PDF 177 kb) [file 12864_2015_1780_MOESM9_ESM.pdf]
