# Supplementary material for: Recombination of chl-fus gene (Plastid Origin) downstream of hop: a locus of chromosomal instability
Source: BMC Genomics. 2015 Aug 4;16(1):573. doi: 10.1186/s12864-015-1780-1 (PMC4522979; doi:10.1186/s12864-015-1780-1)
Supplement: Additional file 4: Figure S3. — Prediction of an intron (dotted vertical line) in Micromonas sp. hop gene (GenBank: XP_002500383), downstream of the first six codons. (A) HCA alignment of the N-terminal amino acids of Micromonas sp. and A. thaliana Hop proteins. Extra 71 amino acids in the Micromonas sp. Hop protein are bordered by a rounded rectangle. Vertical lines connect analogous positions in both proteins. Conserved hydrophobic clusters are gray shaded. Relevant nonhydrophobic identities are indicated by circles on black background. The way to read the sequence and secondary structures, as well as special symbols, are indicated in the inset. (B) Predicted translation of the 5′ regions for Micromonas sp. and C. reinhardtii hop genes. We propose that nucleotides in bold belong to a phase-0 intron, which is in frame with the first and second exons. Splice sites are in italic and underlined. (C) ClustalW alignment [77] of the N-terminal amino acids of predicted Micromonas sp., C. reinhardtii and A. thaliana Hop proteins. The arrow indicates the position of the putative intron Ih in Micromonas sp., and C. reinhardtii hop genes. (PDF 595 kb) [file 12864_2015_1780_MOESM4_ESM.pdf]

A)

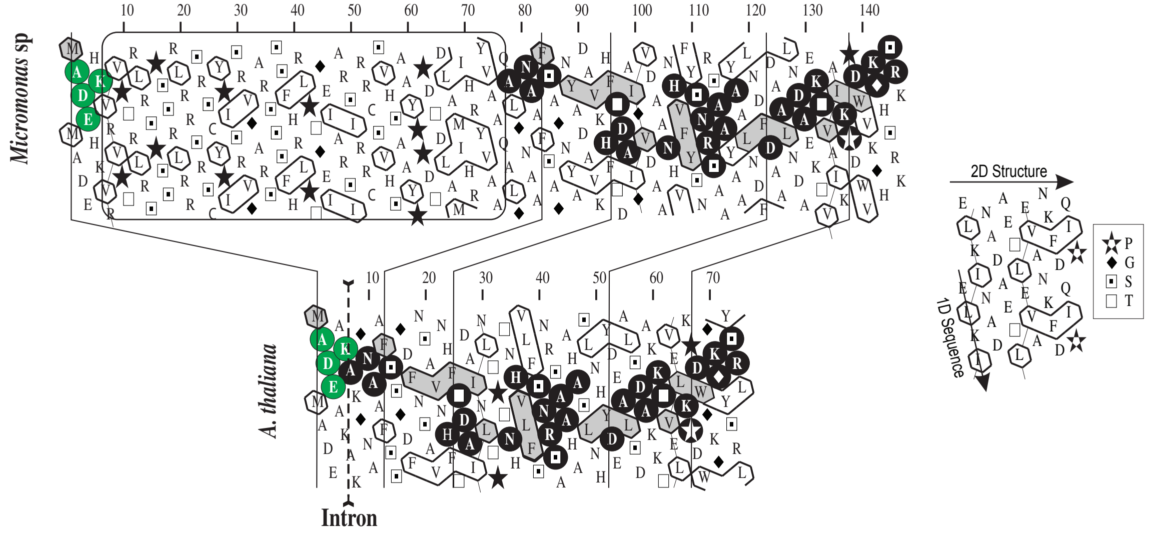

B) *Micromonas* sp.

```
        10        20        30        40        50        60        70        80        90
CGCGTCTGCGCCATTGCGAGCGCCGCAGACGAAGTCCGCCGGAAACGATCGACTCGCCCGCCGCCATGGCCGACGAACACAAGGTGCGCGTCCCGACACG
                                                                 M  A  D  E  H  K  V  R  V  P  T  R

TCTCCGTTCGCCGAGTTCACGCCTCCGCCGCTCGCGATCGTGCTACCCGATCTCGCGCGTCGGCGCCCGCTCGTCTTCCTTCCACCGCCTTCCAACTGGC
 L  R  S  P  S  S  R  L  R  R  S  R  S  C  Y  P  I  S  R  V  G  A  R  S  S  S  F  H  R  L  P  T  G

GAGATCGCGCGTTCGATCCGACGATGTTCCGTCCATTCCGCGGCGTACCCACCCGACACTGACCTCGCGATGATCCACCGATACGTTGCTGCACAGGCTC
E  I  A  R  S  I  R  R  C  S  V  H  S  A  A  Y  P  P  D  T  D  L  A  M  I  H  R  Y  V  A  A  Q  A  L

TCGGCAACGCCGCGTTCAGCGCGGGCAACTACGCCGACGCGGTGAAGCACTTCACCGACGCCATCGGGGTGGACGCCGCCAATCACGTCTTCTACTCGAA
 G  N  A  A  F  S  A  G  N  Y  A  D  A  V  K  H  F  T  D  A  I  G  V  D  A  A  N  H  V  F  Y  S  N
```

Chlamydomonas reinhardtii

```
        10        20        30        40        50        60        70        80        90
GACTAAACCGGGAGGACTGAGCCACGAAGAGCACGTAACCATGTCTTCGGACGAGCTTAAGGTATGTAGCATTCACGATGTCGTAGCGACTGGCAGGGCA
                                        M  S  S  D  E  L  K  V  C  S  I  H  D  V  V  A  T  G  R  A

GCGTGCCGGTATCGAGCAGCACGGAGGGGGCGCCGTCGACGGTGCTGGAAGGCCCGCGCTTACTCCTCGTCACGCACACCCGGTCGCAGGCCAAGGGAAA
  V  P  V  S  S  S  T  E  G  A  P  S  T  V  L  E  G  P  R  L  L  L  V  T  H  T  R  S  Q  A  K  G  N

TGCCGCGTTCAGCGCGGGCAACTTCGAGGAGGCTGCTAAGTTCTTCACGGAGGCAATTGGCGTGGACCCAGGCAACCACGTCCTCTACAGCAACCGCAGC
 A  A  F  S  A  G  N  F  E  E  A  A  K  F  F  T  E  A  I  G  V  D  P  G  N  H  V  L  Y  S  N  R  S
```

C)

Intron

Micromonas sp.	MADEHKALGNAAFSAGNYADAVKHFTDAIGVDAANHVFYSNRSA...
C. reinhardtii	MSSDELKAKGNAAFSAGNFEEAAKFFTEAIGVDPGNHVLYSNRSA...
A. thaliana	MADEAKAKGNAAFSSGDFNSAVNHFTDAINLTPTNHVLFSNRSA...
