# Supplementary material for: Recombination of chl-fus gene (Plastid Origin) downstream of hop: a locus of chromosomal instability
Source: BMC Genomics. 2015 Aug 4;16(1):573. doi: 10.1186/s12864-015-1780-1 (PMC4522979; doi:10.1186/s12864-015-1780-1)
Supplement: Additional file 6: Figure S5. — Hypothetical genes found within the IGR between the hop and chl-fus genes. (A) ClustalW alignment between an inferred pseudogen encoded by the Citrus endogenous (Ce) pararetrovirus (Genbank:KF800044) and the M. notabilis IGR (Mn), found in this work. Colored boxes represent signature domains, including the viral movement protein, zinc finger, reverse transcriptase, and RNAse H. (*): internal stop codons. (B) Three predicted secondary structures [82, 83] of the inverted repeat sequences of a Miniature Inverted–Repeat Transposable Element (MITE) found within the IGR of Oryza spp; dG: Gibbs free energy. (PDF 689 kb) [file 12864_2015_1780_MOESM6_ESM.pdf]

A)

```
Ce     MASTSSSNLSTPLTLSSTLSLPKSCTSHTANKIENLVEYSYIPESAQISESTYPLISPYH 60
Mn     ---METPTQPEPARLSSSISIPSLCKRSLSSRIENLVEYSYIPETAIVKESVPPLLSPYN 57
           .:.. . *  ***::*:*. *.   :.:************:* :.**. **:***:

Ce     LYKRPNSFTRSIRTLISTKRPHPKEYIQSSRLDQCALKATSAEQYVTLEIPSELISNWKR 120
Mn     VFKR---HRRTIRQLISSNKPTIKEYVQSSKLDNCLVPSTSREQYIDLEIPKELITQWLA 114
       ::**    . *:** ***:::*  ***:***:**:* : :** ***: ****.***::*

Ce     EGYTHLHLGGIRLILTLHGRKGLPVTARVALLDTRFKQFQDAVIGTVLTTLHVGSVLLTF 180
Mn     EGHTYLHFGAIRLILTLHGRKGLPVTVRLALLDTRHTKYEHAIIGTTLPTLHASSVLLTI 174
       **:*:**:*.****************.*:******..:::.*:***.*.***..*****:

Ce     YPNFNLSLQDPNLPTTLKVQVQIQGAEQISTAKIATLHHQLVYRLQNHALDLPTPEHHSD 240
Mn     FPNFNIPLKNPNLTTCLKVQLLITGAEQVPSSYIATLHHQLIYRLQNHALDLLVPGHTPD 234
       :****:.*::***.* ****: * ****:.:: ********:**********.* * .*

Ce     TLMVLAESD-QIPTIIQIPRQIPRHELLQLMPLEWISNYEQFHNNTAPVQTSESMFERRP 299
Mn     TLLIIA*RENEIPTIVQIPKQLSREEITKLMPLEWFTNYEKLH*NQKPIHTADPTFIRFE 294
       **:::*  : :****:***:*:.*.*: :******::***::* *  *::*::. * *

Ce     DGTVRMTFKPPPSAPQEPPQLSFTYSSMITAVQTAQEDLPITGFNSDGFLVYPAKQNGHF 359
Mn     DGQVKTVFKFPDENSSTTP------SLMIKPVAT-EEDVPVACFTADGSKIFTDKVDGHF 347
       ** *: .** * . .. .*      * **..* * :**:*:: *.:**  ::. * :***

Ce     LWDVPGSGHCDPDCPCWDDWEEDDDYVTKKKKKPKKKSHASCHHSTPSTPQDPPPPSAPL 419
Mn     IWDSDPS-MCDPDCDCWMDLEEEEDDHPSTRRRKKKGACAPQS*-TTRRQFHPDDPDSP- 404
       :**   *  ***** ** * **::*  ...::: ** : *.    *.    .*  *.:*

Ce     PLYKKELQWLAKRCTPKTSLPVPDPSPPLSCMMFSSASSGYSSSFPPLDTHTDSQRNVVS 479
Mn     --------WAVKKNSRNSSVLDTTP-----CMMFTS----YDQDFPALERQVNQTTKVAS 447
               * .*: : ::*:  . *     ****:*    *...**.*: :.:.  :*.*

Ce     KPFIP-SPITSAGHLEPPKPFESVLNWQTQNARAQ-------------ISLRTEHLETKV 525
Mn     RPYVQPTEIQPDRRIKAPTQAEEVLNWQTQNARAQNQVLQKIETKVDKVIAQANPTDKRL 507
       :*::  : * .  :::.*.  *.************             :  :::  :.::

Ce     DSISAQLQQIHQKLQSRIAQLDSELRTMLAQRYHGPEFDQKEREIRRLKAELAQIES--D 583
Mn     NSLGNEVKRMYENLQSRIKRLDHDLRSLINQGYFGPEFDKKEAEIQKLKAKLK*IYDDLD 567
       :*:. ::::::::***** :** :**:: * *.*****:** **::***:* * .    *

Ce     KQKPSLFTS-SPPIPSIGPTYHPFASMLSPIRQYEPSKLFGMTHTLFRDNPLPSPPKPKP 642
Mn     RRERRMFDPPSYPLPPFGPSLFPSYSYSS---QPDYSKVFKMTHEIFKDLIWSDPSGSKS 624
       :::  :* . * *:*.:**: .*  *  *   * : **:* *** :*:*   ..*. .*.

Ce     KPKPQPRPVTINPSSTIIPDQQSPGYTPASPSPPEPSKSQPSTKSKDKEPMHQFSAHTID 702
Mn     STSTRPTSSKSKTTRMSFSSEEEDEPVSQKPKPAKEKRHEYPHACMVFPSKAERAPESEI 684
       ....:* . . :.:   :..::.   .. .*.*.: .: : .   .    .  : :..:

Ce     HPSSTDDQTSDSNLAVS-DSHTETETESLASTSDSEISYADITRILMAQPD-PQTSRTEP 760
Mn     FSRSASASESDSEPSESPESLPKTETDSKESEEANDT--MDITQLLMATSTGSNDQQTQT 742
       .. *:. . ***: : * :* .:***:*  * . .:    ***::*** .  .: .:*:.

Ce     YVDIPSDVEEEMPESSATNQPPPAQTTSSSQKSSNGPWFTFDDIPSYKWRDRLNEMSAWI 820
Mn     EMGEPTRESSPIVEEPPDTQ*---QSGNTSAKPTIGPWFSFDTVPPDK*REKMSEFGAWI 799
        :. *:  .. : *... .*    *: .:* *.: ****:** :*. * *:::.*:.***

Ce     DLQMLRSGATTESVLREFATRFTGALRDWFDSLGPYRQLQFVQLPEVSSALAILHDQFLG 880
Mn     DLQMSKLRAELQAVLREFSSRFTGTLRFWFQSLGGYRQFQFIKVDTVAGILGLLHQ*FVG 859
       **** :  *  ::*****::****:** **:*** ***:**::   *:. *.:**: *:*

Ce     DPAAAFEAARRDYLSMKCCSLDAKDLDFHYKRMSLLFYKLNGFNEPTLKHVFLASLPEEL 940
Mn     DFNLIDKQIRQEYFEMKCCSLKRKDIEFHYQRMSRQYYMLNGFNDPNLRQVYLSSLPQEL 919
       *      :  *::*:.******. **::***:***  :* *****:*.*::*:*:***:**
```

Viral movement protein

Vps51 super family

```
Ce     QPDIQRQLTASNLVIDNISLGKIFQLAKTCLDKLCEQKQFFKDLLKDKEPFRSACKKPYL 1000
Mn     QPELQRSILATKKKLKDISVGEIHQLALVALNKLCETQELFSSMMKERSKFKKACKKPYL 979
       **::**.: *::  :.:**:*:*.*** ..*:**** :::*..::*::. *:.*******

Ce     QIKCKQKKDCDCSPKKK-RHFRKFKSPEFSSRPRRSRKPYRFFRKRSSSSKDSKRRKSSR 1059
Mn     HIKCKDDK-CFCSPTKHSRDKTTRRSARTKKFDKKEGGRFRFFRKKKHDSP-----KSDR 1033
       :****:.* * ***.*: *.  . :*.. ..  ::.   :*****:. .*      **.*

Ce     CFICRKKGHFAKDCPNKRAKSIRLVEHLQATTDYSPQQ-DELEFYFSEQDEPNDETVFAL 1118
Mn     CFNCGEKGHYSKNCPKKFAKSARIMQQLQASSTYQHEENADLESLYSEQDDPDMDTVFAI 1093
       ** *  :***::*:**:* *** *::::***:: *. ::  :**  :****:*: :****:

Ce     QQS-SDDSDSDQ----SQVIFHQQLLSLDT-TVPIPSIKLQILPSKFQRPIPAIGLIDTG 1172
Mn     PDSDYEDSGPEE*VDYHVPVYNMKQYSMATPTPPLPNIEVQILASKYDNPVNLIAYMDTG 1153
        :*  :**..::       ::: :   *: * * *:*.*::***.**::.*:   *. :***

Ce     AQRSMINPHILPPDSWTQSEEHFKAVNGKLFTTSLITKKPIGIQIFPNCVIWTKVIGSTL 1232
Mn     AGRTMMNLKVLPRDLWIPHIEYFKATDDQVFKTELITKNKISIKFFPGCVIWTKVLGSSL 1213
       * *:*:* ::** * *    *:***.:.::*.*.****: *.*::**.*******:**:*

Ce     PNKDILLGFDILHQIKHLQIIPHGIRVKSMFKPFTDVLKLYNLSETPPSYQDVSTKLLSF 1292
Mn     PDKDLLIGMDVYCQARSLQILPSGLKYKRQFKPYSAILKIFSIKEAPARYEDIKDKLLKC 1273
       *:**:*:*:*:   * : ***:* *:: *  ***:: :**::.:.*:*. *:*:. ***.

Ce     CPESHSEFTHPNPLWKNQSFFVKLPFKLNEDINPTKATHPGMSPSDLLLAQQECSQLLAQ 1352
Mn     CADSHDQFHHPNPL*KNQKFFIQLPFKKNEDVNPTKATHPGMTPSDLALAREECAQLLKQ 1333
       *.:**.:* ***** ***.**::**** ***:**********:**** **::**:*** *

Ce     GLIEPTTSQWACQAFYVEKHSEIVRGKKRLVIDYQPLNMFLQDDKFPLPRRQSMFTFLKN 1412
Mn     GLIEPTRSNWACQAFYVEKRSEQVRGKKMLVIDYKPLNLFLQDDKFPILKTSSLFTHLKE 1393
       ****** *:**********:** ***** *****:***:********: : .*:**.**:

Ce     AQIFSKFDLKSGFWQLGIEPSERYKTAFCIPNAHFQWTVLPFGLKTAPSIFQKSMIKIFQ 1472
Mn     AQVFLKFDLNGAFWQLEIVPGDRPKTAFCIPDAQYQWRVLPFGLKTSPSLFQRTMAQIFE 1453
       **:* ****:..**** * *.:* *******:*::** ********:**:**::* :**:

Ce     PLLSNALIYIDDILLFSGSHDDHHRLLNQFYDIVHSHGIMLSAKKSTIATDTIEFLGMII 1532
Mn     PILYSALIYIDDILLFSKDEEAHKILLAQFHQIVDRHGIMLSEKKSQIGQKQIEFLGMQI 1513
       *:* .************ ..: *: ** **::**. ****** *** *. . ****** *

Ce     KDGHYQPGKHVAQELLHFPDQQLSKKQVQQFLGIINYIRDFIPHVDHYTRHLSALLKKKP 1592
Mn     SQGKFQPSPHIAREL LTFPNKNLTVKQIQQFLGIVNYLRDFIPRVTEHTSQLSKMLKRNP 1573
       .:*::**. *:*:*** **::.*: **:******:**:*****:* .:* :** :**::*

Ce     PEWNDDHTNAVTTLKKIAQNPPPKHQTHPTKKILQTDASDQYWSAVLLEEHNGKRHFCAH 1652
Mn     SQ*GTE*TVAVSALKKIAQSPPPLKIPSTGKRVLQTDASDHYWGAILIEEQDDKKHYCGH 1633
       .:  .  : * **::******.*** :  .  .*::*******:**.*:*:**::.*:*:*.*

Ce     ASGQFKHSEKNYHVIYKEILTVKYGIKKFEFHLISHTFLINMDNSSFPRIFDFKNKLLLD 1712
Mn     ASGQCKESEKHYHTIYKGILAVKYGIQKFDFHLRGFNFEVRMDNSSFPKVLDFKNKTLCD 1693
       **** *.***:**.*** **:*****:**:***   ...* :.*******:::***** * *

Ce     KQLLNLKTWFAKYDFTVQHIKGNQNLIPDFLTRP----------AINKPSLISSIQT--- 1759
Mn     PQLLRLKS*FERYNFSVKHIKGHHNLIPDFLTRPKPVQIITSQKAFPLIYMYNSVHTPLC 1753
        ***.**: * :*:*:*:****::**********          *:    : .*::*

Ce     IPVIALNRQLP----------FKALTQRHFPMNIS-----------------FQSAYQLQ 1792
Mn     FPVKPDHRFSPGLDSTAPPERIKHYARIHLFDYLSRVCKIDPTELMYNPDQPYLAIFHLR 1813
       :** . :*  *          :*  :: *:   :*                 : : ::*:

Ce     DFTKKFL---- 1799
Mn     PFVKVTQDDIX 1824
         *.*
```

Region: zinc finger

Reverse transcriptase

RNAse H

B)

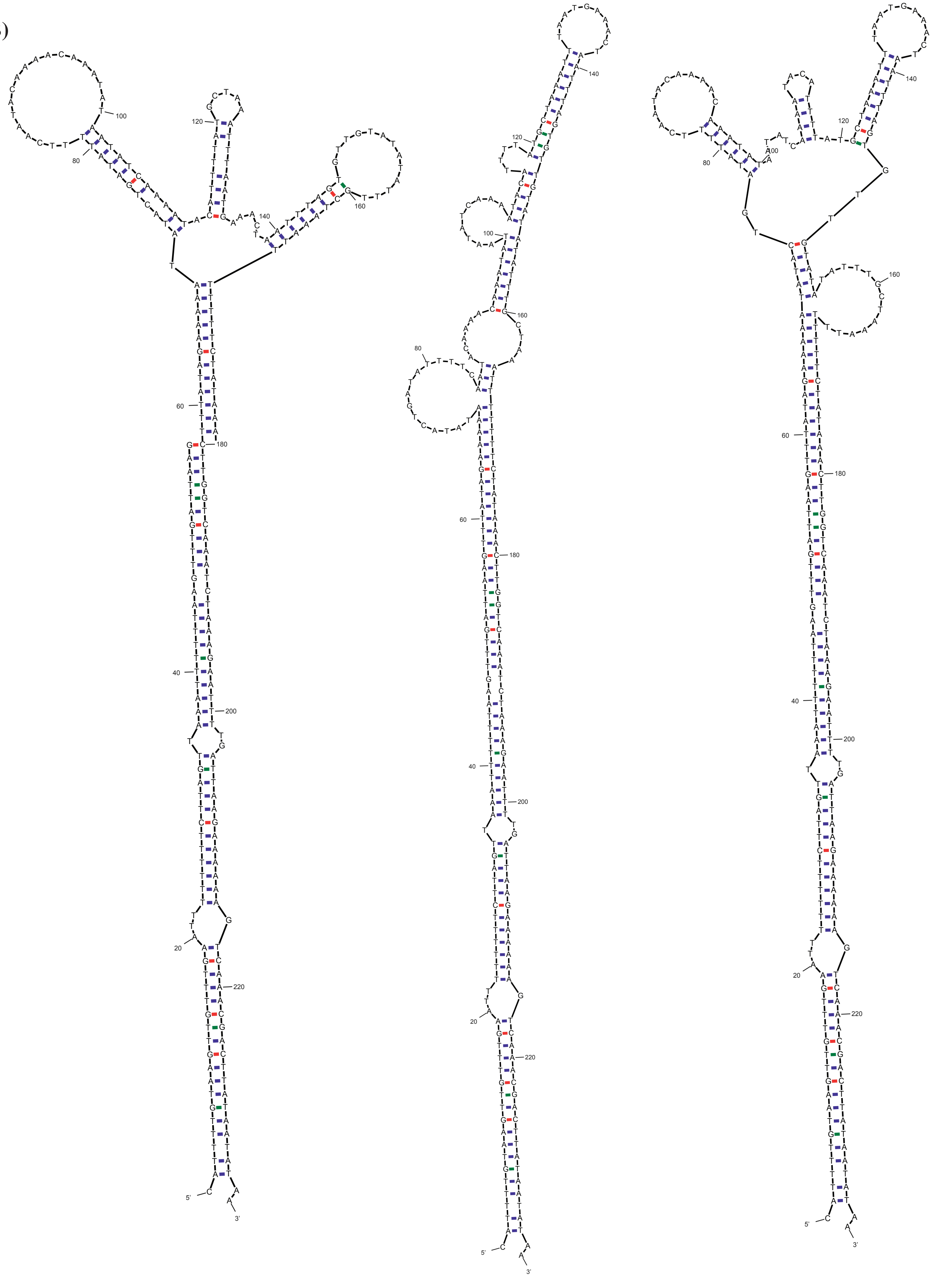

dG = -33.28 Oryza   dG = -33.28 Oryza   dG = -32.03 Oryza
